# AOP-Based Analysis of Curated Transcriptomic Data Reveals a Context-Dependent Core Mechanism of PFAS-Induced Liver Steatosis

**DOI:** 10.1021/acs.est.6c02707

**Published:** 2026-07-30

**Authors:** Nicoletta D’Alessandro, Luca Mannino, Giusy del Giudice, Laura Ylä-Outinen, Noora Perho, Laura A. Saarimäki, Emanuele Di Lieto, Zeyad Al-Abdulraheem, Marcella Torres Maia, Simo Inkala, Lorenzo Campini, Lena Möbus, Jack Morikka, Antonio Federico, Angela Serra, Dario Greco

**Affiliations:** † Finnish Hub for Development and Validation of Integrated Approaches (FHAIVE), Faculty of Medicine and Health Technology, 7840 Tampere University, Tampere 33520, Finland; ‡ Division of Pharmaceutical Biosciences, Faculty of Pharmacy, University of Helsinki, Helsinki 00790, Finland; § Tampere Institute for Advanced Study, Tampere University, Tampere 33100, Finland

**Keywords:** toxicogenomics, data curation, precision toxicology, adverse outcome pathways, per- and polyfluoroalkyl substances, mechanism of action, contextualized mechanisms

## Abstract

Toxicology faces the need to shift from generalized hazard evaluation toward precision approaches that account for the impact of the exposed biological systems. This need is particularly evident for per- and polyfluoroalkyl substances (PFAS), a highly persistent and diverse chemical class whose multiorgan apical toxicities are well documented, yet whose mechanistic understanding remains fragmented. To address this gap, a comprehensive toxicogenomic collection covering multiple PFAS and biological systems is curated, harmonized, and standardized. Through systematic integration of these data with the Adverse Outcome Pathway framework, biological context-aware key event networks that capture the progression from molecular initiating events to apical outcomes are reconstructed. Additionally, new transcriptomic profiles from macrophages exposed to seven PFAS are generated to address the critical but under-investigated immune-related context. Analysis reveals that PFAS toxicity arises from shared early molecular perturbations that diverge across biological systems to produce organ-specific outcomes where immune-related processes consistently emerge as central contributors across multiple contexts. In the liver emerges a conserved mechanistic core underlying PFAS-induced steatosis activated through system-specific pathways shaped by PFAS physicochemical properties and biological context. Overall, this work provides a framework for advancing precision toxicology, enabling rapid, mechanistically grounded, and context-aware PFAS hazard characterization, and supporting the prioritization of uncharacterized PFAS based on shared and context-dependent mechanistic patterns.

## Graphical abstract

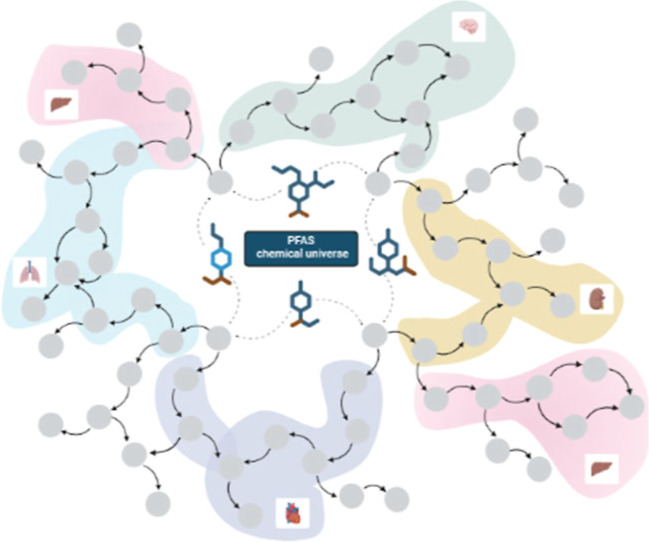

## Introduction

Toxicology has undergone a profound evolution over the past half-century, shifting from phenotype-based observations to mechanistic assessments of chemical hazard.^1^ However, the call for precision toxicology approaches, defined as the generation of contextualized mechanisms of action (MOA) of chemical exposure, is emerging as a necessary evolution of the field. Unlike traditional approaches treating toxicity as an intrinsic property of chemicals, precision toxicology frames it as the outcome of an interaction that depends on multiple dimensions, including chemical structure, exposure duration, and the biological system. Although such contextualized frameworks are well established in other fields, they remain underdeveloped in toxicology,^2,3^ despite growing evidence that molecular composition and physiological complexity fundamentally shape both the initiating and following cascade of events leading to adverse outcomes.^4,5^ Establishing this contextualized relationship is therefore essential for predicting how chemicals perturb specific molecular networks, how they propagate across biological scales, and why adverse outcomes manifest in specific contexts.

The need for contextualized approaches is particularly critical for PFAS, synthetic chemicals whose exceptional chemical stability and resistance to heat, water, and oil have driven their extensive use in thousands of industrial applications and consumer products.^6^ However, these same properties drive their extreme environmental persistence, resistance to degradation, and propensity for long-term bioaccumulation,^7,8^ raising substantial concerns regarding their toxicological impacts on both human health and the environment.^9,10^ As a result, several epidemiological and experimental studies have linked PFAS exposure to a broad variety of adverse health outcomes.^9,11–13^ Among them, metabolic disruption and hepatic toxicity are the most frequently reported, with multiple studies linking liver damage with lipid metabolic disorders caused by PFAS exposure.^14,15^ Despite their well-characterized health effects, PFAS toxicity data are often associative, with limited understanding of the mechanistic events that link exposure to apical adverse effects. This lack of mechanistic resolution is especially problematic given the multisystem nature of PFAS toxicity: they affect several organs, indicating that toxicity emerges not solely from the intrinsic properties of the chemicals but from their interaction with specific biological systems. This mechanistic gap aligns with the limitations of the current chemical safety assessment framework, which still relies heavily on chemical-by-chemical, phenotypic-based testing. Such approaches are too slow and costly to evaluate the nearly ten thousand PFAS compounds currently in commerce within a realistic time frame.^16^ Consequently, most of PFAS remain uncharacterized, and their toxicity assessment remains retrospective, relying on epidemiological evidence to detect adverse health associations only after widespread human and environmental exposure has occurred. Addressing these limitations requires integrative, mechanistically grounded, and biologically contextualized approaches capable of resolving PFAS toxicity across chemical diversity and biological scales.

Toxicogenomics has provided molecular level characterization of chemical perturbations and expanded our view of PFAS toxicity molecular MOAs such as those extensively reviewed here.^17^ However, transcriptomic responses alone rarely translate into mechanistic predictions of the apical outcomes. Conversely, the Adverse Outcome Pathway (AOP) framework formalizes causal relationships at the multiscale level, from an MIE to an AO through key events (KE), but in its current form remains disconnected from molecular data.^18^

To bridge this gap, we previously developed a gene-level annotation of human-relevant AOPs, enabling direct integration of toxicogenomic data within mechanistic AOP networks.^19^ Building on this, we retrieved, harmonized, and standardized publicly available PFAS toxicogenomic data sets. While most existing data derive from liver models, increasing evidence shows that PFAS also disrupt immune function, altering cytokine signaling, immune-cell activation, and reducing antibody responses to vaccination, prompting regulatory agencies to identify immunotoxicity as a critical end point for PFAS risk assessment.^20–23^ To address this underrepresented biological context, we complemented the public data sets by generating transcriptomic profiles from macrophages exposed to seven PFAS, including both legacy and newer-generation compounds.

By embedding this curated collection within an AOP-based system-aware framework, we reconstructed PFAS mechanisms in a context-dependent manner, highlighting how chemical structure and biological system jointly shape the progression from MIEs to apical AOs. Through this strategy, we advance PFAS hazard characterization toward precision toxicology, supporting faster, mechanistically grounded, and contextualized prioritization across the PFAS chemical space.

## Material and Methods

### Data Collection and Manual Quality Assessment

To compile a comprehensive collection of PFAS-related transcriptomic data sets, we searched three public repositories: Gene Expression Omnibus (GEO), ArrayExpress, and Zenodo.^24–26^ Repositories were queried using the keywords “PFAS” and “Per- and Polyfluoroalkyl Substances”. Since all entries identified through ArrayExpress and Zenodo overlapped with GEO records, only GEO data sets were retained in subsequent steps (latest query September 2024).

The initial search returned 65 unique entries, which were reduced to 32 by restricting selection to transcriptomic data sets generated in the most used and human-relevant species (human, mouse, rat, and zebrafish). Data sets were then subjected to manual quality assessment. Transcriptomic data quality depends not only on the underlying technology but also on the rigor of the experimental design, execution, and the completeness of associated metadata.^27^ To ensure reliability and compatibility across studies, we applied the following inclusion criteria adapted from those used in prior toxicogenomic data collection:^28^

- Availability of raw data: Access to raw data enables consistent preprocessing across data sets using a unified analytical pipeline.
- Detailed metadata: Data sets had to include sufficient experimental information (e.g., treatment conditions, doses, time points, biological models, replicates) to support accurate preprocessing and interpretation.
- Presence of an untreated reference group: A reference condition is mandatory to allow robust estimation of treatment-induced transcriptional changes.
- Inclusion of at least three biological replicates: Although the required number of replicates for sufficient statistical power varies by biological question, experimental system, and technology, a minimum threshold of three replicates was set to ensure a baseline level of reliability.

Each entry was manually evaluated against these criteria. Twenty out of 32 data sets satisfied all requirements and were retained for subsequent steps. In cases where only specific experimental groups within an entry failed to meet inclusion criteria, those were excluded while retaining the remaining entries (Table S1). Additionally, only experimental groups corresponding to biologically relevant sublethal exposure doses were included. Experimental conditions reported as overtly toxic in the original studies were excluded from the analysis. To ensure full interoperability, the newly generated data set on PFAS-exposed macrophages was added to the pool of data sets that satisfied the manual quality assessment and analyzed with the same approaches (Supporting Information IV includes details on experimental design, exposure, and sequencing).

### Metadata Curation

To curate and harmonize metadata before data preprocessing, we used ESPERANTO, a recently developed R/Shiny application that supports toxicogenomic metadata curation in alignment with Good Laboratory Practice principles.^29^ For each data set that passed manual quality assessment, metadata were retrieved using the getGEO function from the GEOquery R package (v2.7.00).^30^ All curated and harmonized metadata files are included in the final data collection.

### Data Preprocessing

The data collection comprising 9 DNA microarray data sets, 9 RNA-seq data sets, and 2 TempO-Seq Detailed preprocessing workflows is described in Supporting Information IV.

### Data Set Harmonization and Exposure Period Grouping

Prior to downstream analysis, DEGs identified from each comparison that originate from *Mus musculus*, *Rattus norvegicus*, and *Danio rerio* model species were converted to human Ensembl IDs by using orthologous gene mapping data obtained from biomaRt.^31^ This step was essential to enable cross-species comparison and to support the derivation of human-relevant mechanistic insights, although it may obscure species-specific transcriptional responses. TempO-Seq probe identifiers were mapped to Ensembl IDs by using biomaRt (v2.40.1; GRCh38.p14, Ensembl release 114).

The 388 exposure studies of the collection were grouped based on exposure duration. Three exposure periods have been defined, titled short, intermediate, and long with different intervals for in vivo and in vitro studies as previously done.^32–34^ In short, based on Organisation for Economic Cooperation and Development (OECD) guidelines in vitro studies were divided into short, intermediate, and long exposure duration based on thresholds of 24 h, 72 h, and longer than 72 h. While in vivo studies were divided into short, intermediate, and long exposure duration based on thresholds of 72 h, 720 h, and longer than 720 h (Figure S2).

### KE Enrichment Analysis, AOP Fingerprints Extraction and KE–KE Relationship Network Mapping

DEGs identified for each exposure study were used as input for enrichment testing against the AOP and KE gene annotation curated by Saarimaki et al.^19,35^ Enrichment was performed independently for each exposure study using the AOPfingerprint package (https://github.com/fhaive/AOPfingerprintR) and BMDx2 tool.^35^ The background for enrichment testing comprised all the genes measured within the platform used to generate data. AOP and KE were considered significantly enriched at FDR-adjusted *p*-value <0.05. To mitigate bias arising from heterogeneity in transcriptomic platform coverage, significant AOPs and KEs were ranked by adjusted *p*-value to retain the most statistically supported perturbations. A mean-based cutoff was then applied to standardize the number of enriched entities across all exposure studies, improving comparability between data sets. AOP fingerprints were subsequently defined for each exposure study as those AOPs significantly enriched for which at least 66% of their constituent KEs were also significantly enriched. This threshold was predefined on the premise that, within the AOP framework, the likelihood that a pathway is meaningfully engaged increases with the proportion of its constituent KEs concurrently altered. Requiring a two-thirds majority therefore prioritizes AOPs with coherent, multievent perturbation profiles over those supported by isolated or fragmented signals, strengthening confidence in the inferred progression toward an AO. A sensitivity analysis confirming that this threshold yields more connected and mechanistically complete AOP profiles is provided in Figure S4. Being the full AOP and KE enrichment valid and useable for interpretation, they have been made available in the associated repository. AOP fingerprints were then aggregated by exposure period, and their unique KEs were mapped onto directed KE–KE networks using the edge lists retrieved from the AOP-Wiki database (downloaded 10 June 2022). To obtain more complete mechanistic representations, we incorporated nonenriched KEs using the completeness approach described in,^4^ which applies the shortest.paths function in the igraph R package to connect each enriched KE to its two closest MIEs and AOs, with a maximum path length of 5.^36,37^ Networks were visualized using the VisNetwork package.^36,37^ Because figures in the manuscript are reconstructions^38,39^ generated in BioRender, the original interactive network files (HTML format) and the codes used for generating them are provided for transparency and reproducibility.

### KEs Refinement of PFAS-Induced Liver Steatosis Mechanisms

To improve the interpretability of the PFAS^40,41^ -induced liver steatosis subnetwork, we merged redundant KEs describing equivalent biological processes. Redundancy, a known challenge in the AOP framework, was defined as the occurrence of KEs that were semantically similar and shared highly overlapping gene sets.^38^ First, all AOPs associated with liver steatosis were retrieved from the AOP–gene set annotation developed by Saarimäki et al.^19^ We extracted KEs included in these AOPs and retained only those that were significantly enriched in the KE enrichment analysis (KEs = 24). To identify redundant KEs, pairwise gene set similarity was computed using the Jaccard Index normalized by the size of the smaller gene set to reduce bias arising from unequal gene set sizes. KEs showing both high Jaccard similarity and semantic equivalence of their biological description were merged­(Figure S7). Based on this approach, following events were combined: Event:860 (Decreased, Mitochondrial Fatty Acid Beta Oxidation) with Event:179 (Decrease, Fatty acid beta-oxidation) and Event:1424 (Reduced, fatty acid beta oxidation); Event:459 (Increased, Liver Steatosis) with Event:1418 (increased, steatosis); Event:231 (Decreased, PPAR-alpha activation), Event:232 (Decreased, PPAR-beta activation), Event:233 (Decreased, PPAR-gamma activation), and Event:1422 (Reduced, PPAR-alpha); Event:291 (Accumulation, triglyceride) with Event:454 (Increased, Triglyceride formation). Merged events retained the original name or were assigned a composite name reflecting their shared biological meaning. To avoid loss of mechanistic information, all incoming and outgoing causal connections of the merged events were maintained. The resulting network was visualized in Biorender.

## Results and Discussion

### Building a High-Quality, Interoperable Toxicogenomic Data Collection for PFAS Precision Toxicology

To investigate the contextualized mechanisms of PFAS toxicity, we retrieved and curated from public repositories available transcriptomic datasets generated across diverse biological systems and PFAS chemistries. A main challenge in toxicogenomics is the poor comparability across datasets, which hinders the ability to draw robust, integrative conclusions. Although data-sharing frameworks such as the FAIR principles aim to make data Findable, Accessible, Interoperable, and Reusable, omics datasets still vary widely in experimental design, processing, and metadata reporting.^42–44^ Beyond these, omics data also vary in the technologies used to generate them.^27,28,42,44–46^ To account for these variabilities and robustly interpret the output data, we first applied a set of quality inclusion criteria to the 32 retrieved entries. These criteria were defined to retain only data sets with appropriate experimental design that allowed for reliable extraction of exposure-related transcription alterations, specifically, those including appropriate control groups and sufficient biological replicates to enable robust statistical analysis. Following manual assessment, 12 entries were discarded (Table S1). Their interoperability was then improved by metadata harmonization, making their vocabulary consistent and machine readable, and by preprocessing each entry applying standardized pipelines.^29,47^ Our collection spans three technologies, each with distinct chemistry and gene coverage. These intrinsic differences introduce variability in sensitivity and dynamic range, potentially biasing downstream analysis.^48^ To mitigate these effects and ensure cross-study interoperability, all data sets underwent standardized preprocessing pipelines tailored to each technology. A summary of the entries included in the collection is reported in Supporting Information I. This resulted in a transcriptomic collection of 388 exposure studies. Overall, the collection comprises 29 PFAS molecules tested across 9 biological systems from 4 species (Figure 1, Table S2). A detailed exposure study table is reported in Supporting Information II.

**Figure 1:**
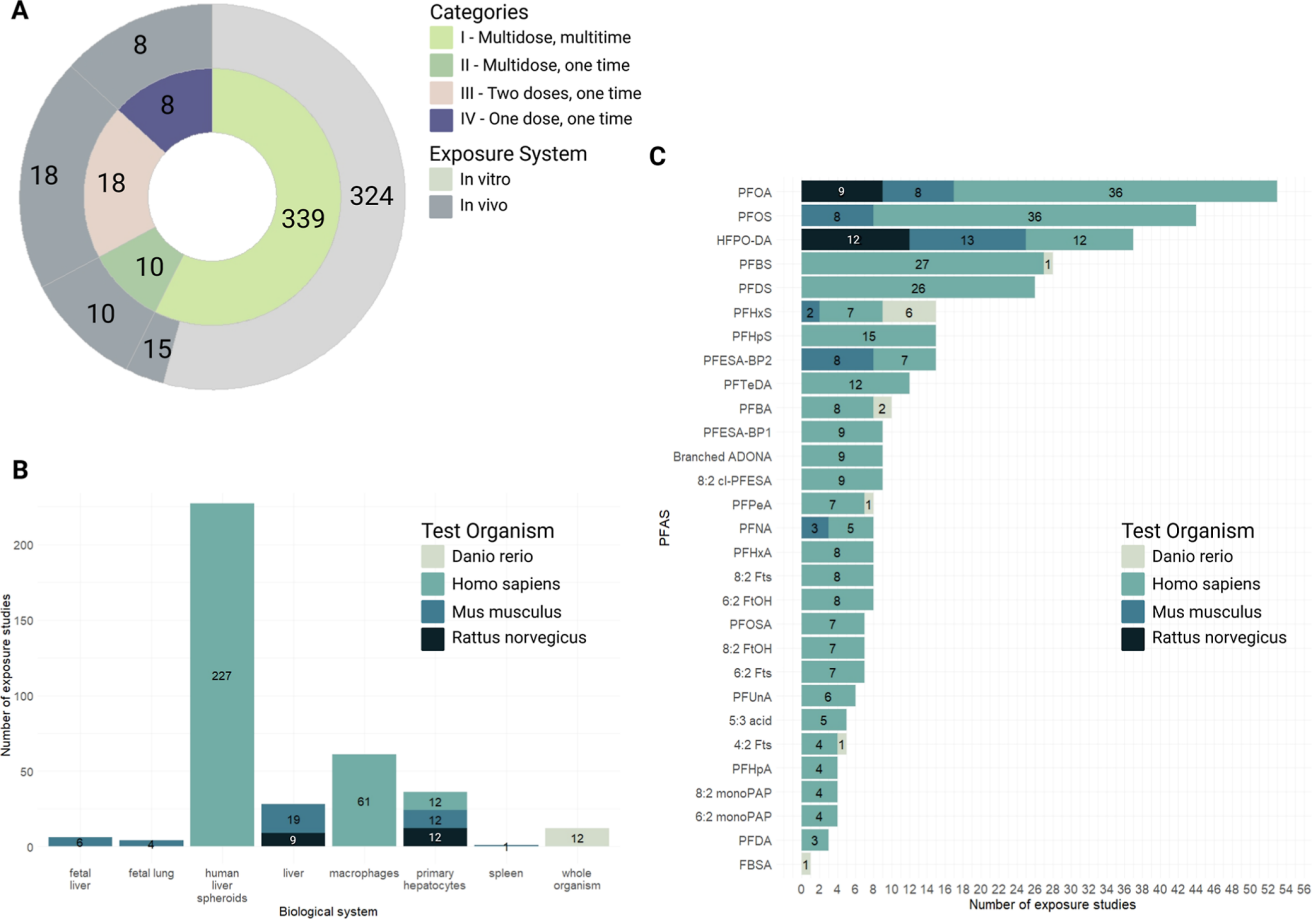
Characteristics of the 388 exposure studies included in the curated PFAS data collection. (A) Donut chart representing studies categorization in four classes based on doses and time point tested (inner ring) as well as on the exposure system (outer ring). (B) Bar plot representing the distribution of exposure studies across biological systems. (C) The bar plot represents the distribution of exposure studies across PFAS tested. In (B) and (C) each bar is colored according to the test organism in which PFAS have been tested.

Legacy PFAS, particularly PFOS and PFOA, constitute the largest portion of the collection, consistent with decades of investigation due to their persistence, bioaccumulation, and well-documented human health outcomes (Figure 1C).^49^ HFPO–DA, a replacement for PFOA, is also well represented, reflecting growing concern regarding its comparable toxicological profile.^50,51^ PFNA and PFHxS were also tested in a moderate number of studies. In contrast, long-chain PFAS (e.g., PFHpA, PFDA, PFDS) and emerging alternatives (e.g., monoPAPs, FTOHs) remain underrepresented, despite increasing evidence of potential toxicity.^52^ This imbalance underscores the need for approaches that generalize mechanistic insights across the PFAS class, enabling extrapolation beyond compound-specific effects.

Liver-based models dominate the collection, including primary hepatocytes, human liver spheroids, and liver tissues (Figure 1B). Although represented only minimally, fetal liver tissue further completes this hepatic context. As illustrated in Figure S1, only a limited number of PFAS have been tested in nonliver systems. This predominance reflects the central role of the liver in PFAS accumulation and toxicity^10,53^ and indicates that the collection is well-suited for deriving mechanistic insights within hepatic systems. At the same time, it underscores a strong liver-centered focus in the current data landscape, potentially overlooking other biologically relevant compartments and highlighting the need for further systemic characterization. Despite substantial epidemiological evidence linking PFAS exposure to reproductive dysfunction and immune dysregulation,^54^ these biological contexts remain largely absent from publicly available transcriptomic data, leaving critical mechanistic questions unresolved. To partially address this gap, we generated transcriptomic data in an in vitro model of monocyte-derived macrophages (THP-1 cell line) exposed to seven PFAS at multiple concentrations and time points (Supporting Information IV). Once integrated into the collection, macrophages became the second most represented biological system, improving coverage of immune-relevant contexts. Additional systems present in the data set, such as zebrafish embryos, fetal lung, and spleen, provide further biological diversity and underscore areas where additional molecular studies are needed. To facilitate possible downstream analysis, each exposure study was categorized according to the combination of doses and time points tested (Figure 1A). Most of the studies fell into Category I and Category II, which include experiments testing multiple doses across multiple or one time point, respectively. Previous studies have shown the effectiveness of multidose and multitime toxicogenomic data analysis for the characterization of chemicals MOA.^55–57^ Multidose testing is essential in toxicology to derive dose-dependent results, often regarded as the direct effects of chemical exposure.^58^ Since majority of the collected exposure studies include multidose experiments, our data collection can be used for modeling dose-dependent changes associated with PFAS exposure.

While heterogeneity across studies cannot be fully resolved, this curated collection represents a high-quality, interoperable resource that balances inherent data heterogeneity with methodological consistency. Although the collection is skewed toward legacy PFASs and liver systems, it provides a robust foundation for deriving contextualized mechanisms in well-studied tissues while revealing missing contexts where mechanistic evidence is needed. All curated data sets, harmonized metadata, normalized/raw count matrices, and differential expression results are available on 10.5281/zenodo.17985509 as a reusable, extensible community resource.

### Toxicogenomics Data and AOP Integrated Approach Reveals Molecular Mechanisms Underlying the Broad Toxicity Spectrum of PFAS

Given the richness and interoperability of the compiled PFAS transcriptomics data collection, we leverage it to address the limited knowledge of the mechanisms linking molecular perturbations to AOs. Therefore, we analyzed the collected omics data within the context of the AOP framework. Because the exposure duration shapes toxicological responses, temporal context was incorporated in our analysis. Thus, we grouped the exposure studies into short-, intermediate-, and long-term exposure periods, enabling comparative analysis across different exposure durations. Time categorization (Figure S2) was performed separately for in vitro and in vivo systems, as previously described in refs 32,33 As a result, we obtained 135, 97, and 156 exposure studies at short-, intermediate-, and long-term exposures, respectively (Figure S3A). For each exposure study in each exposure period, we performed KEs and AOPs enrichment analysis on their DEGs and extracted AOP fingerprints. This approach identified 65 KEs at short, 101 KEs at intermediate, and 39 KEs at long-term exposure periods.

To explore the human health hazards associated with PFAS, we mapped the 42 uniquely enriched AOPs identified across exposure durations to OECD SSbD human health hazard categories.^59^ These AOPs spanned seven human health hazard categories consistent with known PFAS associated toxic end points^60–62^ (Figure 2).

**Figure 2:**
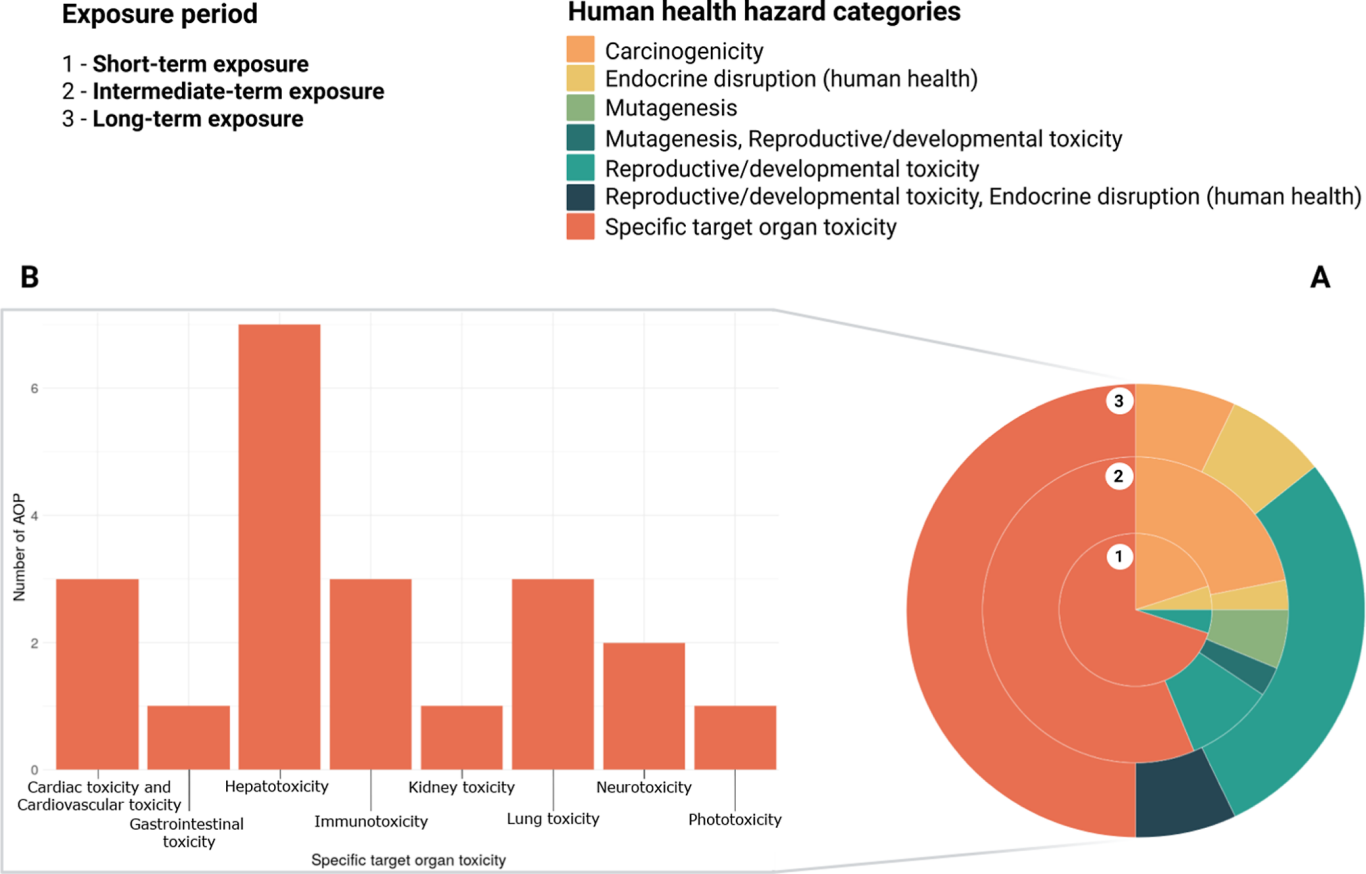
Health hazard categorization of AOPs. (A) Multilayer pie chart showing the proportion of AOPs associated with each health hazard category. Each color corresponds to a specific health hazard category, while each ring represents a different exposure period. (B) Bar plot showing the number of AOPs linked to the end points included in the Specific Target Organ Toxicity category across exposure period.

Except for mutagenesis, which appeared only at intermediate exposures, the other categories were consistently affected across exposure durations, though with varying magnitude. Reproductive/developmental toxicity and specific target organ toxicity increased in prevalence with longer exposures, suggesting temporal dependency of PFAS mechanisms (Figure 2A). In contrast, carcinogenicity category decreases with exposure durations, despite PFAS being classified as potential carcinogens.^62–64^ However, AOPs reduced representation at long durations may reflect experimental biases more than a recovery of acutely activated carcinogenic signature: most long-term studies in our data set relied on the TempO-Seq platform, which targets approximately 2000 liver-related genes.

Despite PFAS being broadly classified as endocrine disruptors, only AOP:69 “Modulation of Adult Leydig Cell Function Subsequent to Decreased Cholesterol Synthesis or Transport in the Adult Leydig Cell” was consistently enriched across exposure durations. This AOP, culminating in Event:646 “Decreased Cholesterol, Decreased sperm quality and/or quantity in the adult testis”, was fully enriched across exposure periods. Epidemiological studies have linked elevated PFOS/PFOA levels with reduced sperm count and testosterone levels, while rodent studies associate impaired sperm quality to PFAS-induced disruption of PPAR signaling in Leydig cells.^65,66^ However, AOP:69 lacks key event relationships (KERs). Given the AOP theoretical nature that often connects events very distantly on a temporal and spacial scale, a missing causal link may be envisioned through indirect mechanistic connections (Figure S5). Additionally, we observed enrichment of PPAR-related events outside AOP:69. Considering that PPAR signaling regulates cholesterol synthesis and transport, PFAS-induced perturbation of PPARs pathways offers a plausible mechanistic bridge connecting upstream molecular disruption to the MIEs of AOP:69, ultimately leading to adverse reproductive effects. This interpretation is consistent with reported PFAS effects on Leydig and adrenal cells.^66^ Although endocrine-specific systems were not directly represented in our data set, transcriptional alterations detected in liver systems offer indirect mechanistic evidence relevant to testosterone-producing cells. In this context, our findings suggest a biologically plausible mechanistic link between PPAR modulation and cholesterol-related events within AOP:69. This observation generates hypotheses regarding potential connections between upstream and downstream events. However, the formal establishment of KERs would require a dedicated Weight of Evidence assessment beyond the scope of this study. “Specific target organ toxicity” was the largest category enriched across exposure periods involving eight organ-specific end points (Figure 2B). This broad activation pattern underscores the need for context-dependent interpretation of PFAS molecular effects, as the same upstream perturbations can manifest differently across biological systems. Among these end points, hepatotoxicity was the most frequently altered, consistent with the liver being a primary site of PFAS accumulation, metabolic disruption, and lipid dysregulation,^67^ followed by cardiovascular, lung, and immunotoxic effects.

Taken together, these findings reinforce the view of epidemiological observations of the PFAS class as a multisystemic toxicant. This systemic pattern highlights the importance of investigating mechanistic pathways across tissues to fully capture the health impact of PFAS exposure.

### PFAS-Induced Liver Steatosis Arises from Convergent but Biological System-Specific Mechanisms

Hepatotoxicity is the most well-documented adverse health outcome associated with PFAS exposure.^62^ Our toxicogenomic collection includes a wide set of PFAS tested on hepatic models spanning across multiple levels of biological complexity. This diversity is advantageous, as simpler systems may better resolve early molecular perturbations, whereas complex systems may capture apical events closer to the AO. From the biological system point of view, this makes our data collection a strongly relevant set of studies to investigate liver relevant mechanisms. Accordingly, our analysis identified hepatotoxicity as the most frequently activated targeted-organ end point (Figure 2B) and within it, Event:459 “Increased, Liver Steatosis” emerged as most frequently enriched, which represents a relevant end point of PFAS toxicity.^15^ To address the lack of precise mechanisms linking metabolism disruption to PFAS-induced liver injury,^17^ we investigated the mechanistic basis of PFAS toxicity end points by mapping the identified KEs onto the KE–KE network, thereby interpreting the results within an established AOP-based causal framework. This network-level approach moves beyond individual AOPs, enabling identification of mechanistic connections between events that are not captured within single AOPs, thereby supporting a more complete interpretation.

On the liver steatosis subnetwork (Figure S6), we identified five distinct MIEs: PPARs deregulation “activation of glucocorticoid”, “PXR receptors”, “Nrf2 repression”, and “systemic inflammation”. These results suggest that PFAS-induced liver steatosis starts as a NR modulation paired with an inflammatory response. These initial MIEs subsequently propagate through interconnected metabolic and inflammatory processes, converging on a common set of events suggesting that PFAS exposure can induce liver steatosis through multiple complementary pathways.

Since AOPs are developed independently, redundancy of KEs across pathways is common. We also observed this redundancy in the PFAS-induced liver steatosis subnetwork. To improve the interpretability of the mechanism, we merged semantically redundant KEs with shared similarity of their annotated gene sets (Figure S7). The resulting network provides a biologically coherent PFAS-induced liver steatosis mechanism, preserving its mechanistic integrity (Figure 3).

**Figure 3:**
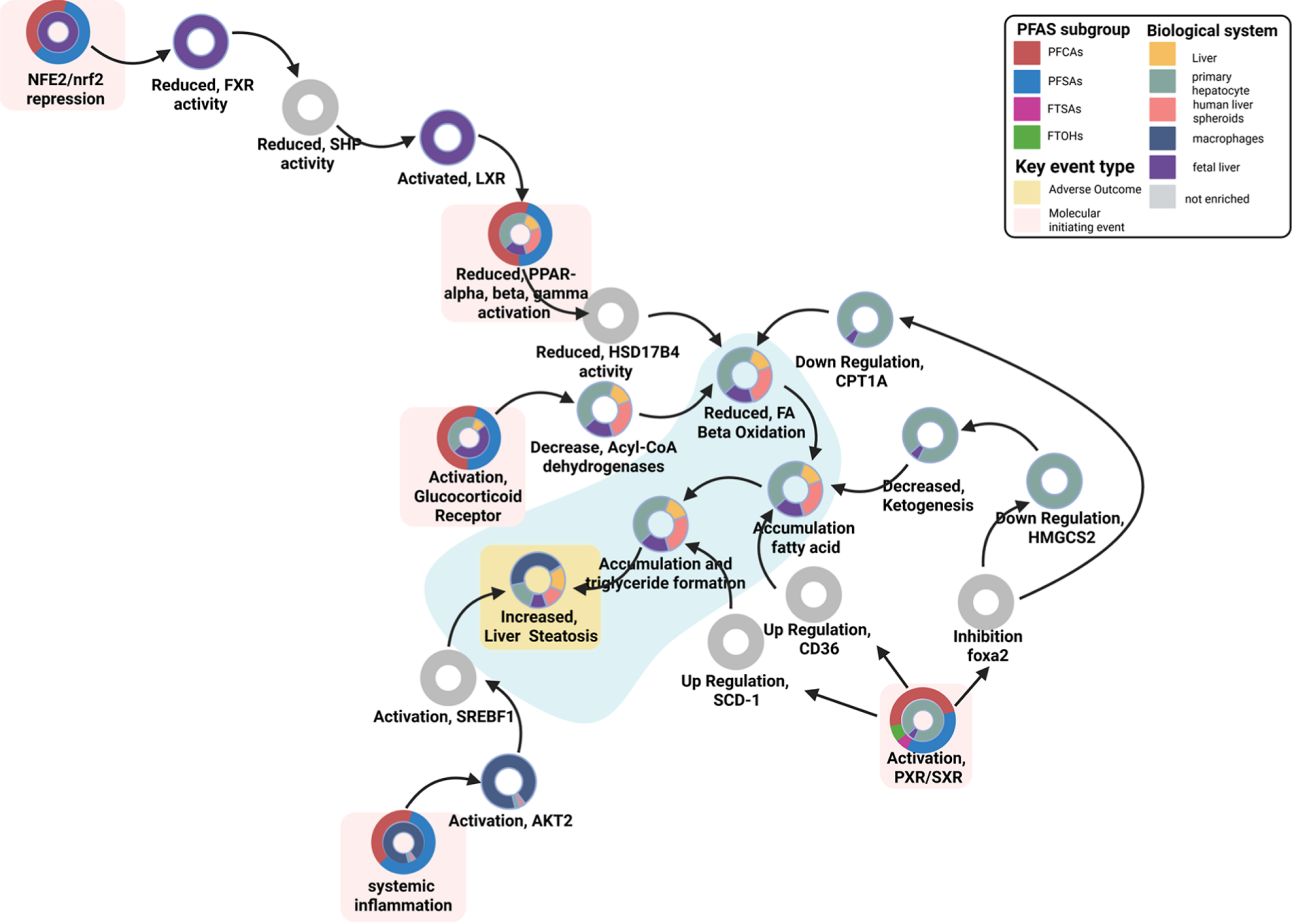
PFAS-induced liver steatosis mechanism subnetwork. This figure depicts the summarized subnetwork KEs and KERs underlying PFAS-induced liver steatosis. Each KE is visualized as a donut chart, where the relative proportions represent the distribution of biological systems in which that KE was enriched across all available data sets. The background square color denotes the KE type while the blue cloud underling the set of events considered the core mechanism of PFAS-induced liver steatosis. For MIEs, the additional outer ring indicates the distribution of PFAS subgroups contributing to the enrichment of that specific MIE.

To contextualize the mechanistic chain leading to liver steatosis, we next evaluated whether specific KEs were primarily driven by the biological system or by PFAS. For this purpose, we annotated KEs in the network by the biological system and the PFAS compound that enriched it, enabling us to resolve system-specific and chemical-specific contributions to the mechanism (Figure S6). Network annotations by individual PFAS revealed no mechanistic branches uniquely associated with PFAS molecules (Figure S6A). Instead, the overall mechanism was activated across exposure periods by PFOA, PFOS, HFPO–DA, and PFNA. A few PFAS selectively activated inflammatory events most likely reflect limitations in data availability rather than genuine compound-specific effects on inflammation (Figure S1). The same limitations in data availability may also explain the absence of AOP fingerprints for the emerging PFAS. Whereas legacy PFAS have been tested across multiple biological systems and exposure durations, emerging PFAS are represented in a few systems and often assessed using lower-coverage transcriptomic technologies (Table S2). Consequently, the lack of AOP fingerprints in our analysis likely reflects insufficient data to meet fingerprint thresholds rather than a true absence of toxicological effects, as supported by existing in vitro evidence linking emerging PFAS to hepatotoxicity.^68^ To examine this, complete inspection of KE enrichment profiles revealed that emerging PFAS, at longer exposure durations, enriched PPAR and PXR activation, indicating engagement of the same upstream pathways as legacy compounds but with delayed kinetics. As NRs activation requires intracellular PFAS accumulation, differences in cellular uptake likely explain these temporal patterns.^69,70^ Supporting this hypothesis, docking analyses revealed comparable predicted binding affinities of legacy and emerging PFAS toward all NRs examined (Figure S8), indicating that emerging PFAS retain the molecular capacity to initiate the same mechanistic cascades. These results are consistent with the hypothesis that delayed activation may reflect differences in intracellular availability rather than a reduced receptor-binding potential. However, docking alone does not demonstrate receptor activation or downstream biological effects, and targeted studies are required to confirm these observations.

In contrast, biological system annotation (Figure S6B) revealed clear system-specific mechanistic branches including a branch almost uniquely activated in macrophages, a distinct pathway specific to fetal liver, and a larger portion including three branches, restricted to adult liver systems. Our results show that different systems may activate distinct mechanistic routes converging on the same AO. These findings indicate that mechanistic specificity is more clearly resolved across biological systems in which the perturbation is measured than across individual PFAS compounds, with the activation of KEs reflecting their functional specializations and levels of biological organization. Moreover, they underscore the importance of accounting for system-specific contributions in risk assessment, as advocated by a growing body of literature.^4,5^ In summary, our results reveal that PFAS exposure may activate system-specific mechanistic branches converging into a shared core of events leading to liver steatosis. Despite this specificity, as pathways progress toward the AO, a common set of KEs appear to be shared across biological systems (Figure 3). This pattern indicates that PFAS can trigger diverse molecular perturbations across systems, that ultimately converge on a potentially conserved mechanistic core associated with liver steatosis. It should be noted that the KEs and KERs constituting this mechanism derive from AOPs currently classified as “none” in AOP-Wiki, indicating that they have not yet undergone formal OECD external review (Table S3). This status mirrors the broader landscape of AOP-Wiki (Figure S9) and should therefore be interpreted as reflecting the current developmental state of the AOP framework rather than as a limitation of this study. Accordingly, the proposed mechanism should be understood as a hypothesis-generating framework rather than a regulatory-ready assessment. However, the biological plausibility of identified mechanistic chains is independently supported by literature, which provides confidence in the proposed context-dependent interpretation beyond formal OECD endorsement status.

### Preferential Activation of MIE of the PFAS-Induced Liver Steatosis Core Mechanism Is Context-Dependent

KE–KE network analysis revealed a system-agnostic mechanistic core initiated through different pathways depending on the biological system. Within the liver-related portion of the mechanism, PFAS exposure appears to activate multiple mechanistic branches simultaneously, each initiated by a distinct MIE (Figure 3). While many studies that investigate PFAS disruption of lipid metabolism showed that the liver injury is primarily mediated through modulation of PPAR-alpha,^14,17,71^ others including knockout screens in liver mice, indicated that PPARs signaling alone does not fully explain PFAS-induced liver steatosis, suggesting the involvement of other NRs mediated mechanisms.^15,72^ Thus, it is still unknown which molecular targets play the central role in triggering liver steatosis.

Our results support the hypothesis that activation of NR-specific branches within liver steatosis mechanism depends on the biological system, with tissue-specific NR expression profiles therefore potentially prioritizing alternative mechanistic routes toward the same AO. Exploration of NR gene expression in embryonic and adult human and mouse tissues on Tabula Sapiens^73^ showed that PPARα and FXR are most highly expressed in fetal mouse hepatocytes, whereas PPARs predominate in adult human liver. In adult mouse liver, NR expression levels are comparable. These differences may explain why NRF2-related mechanisms appear preferentially engaged in fetal tissues, while adult tissues rely more heavily on PPAR-mediated pathways, without necessarily indicating a unique central initiating event. Consistent with this interpretation, we next examined whether PFAS structural features influence preferential MIE activation. While the liver steatosis mechanism was predominantly driven by legacy PFAS, emerging PFAS mainly affected MIEs at longer exposure durations (Figure S6A). Multiple strategies for grouping PFAS are in use, among the most common the classification by headgroup is particularly informative, as structural features determines physicochemical behavior, bioaccumulation, and molecular interactions.^74^ Thus, we grouped PFAS according to headgroup chemistry resulting in six subgroups (Table S4), with perfluorocarboxylic acids (PFCAs) and perfluorosulfonic acids (PFSAs) being the most represented. Grouping the PFAS revealed that, although some compounds activated multiple MIEs, PPAR receptors were preferentially activated by PFCAs, while PXR was more strongly engaged by PFSAs and selectively activated by fluorotelomer alcohols (FTOHs) and fluorotelomer sulfonic acids (FTSAs) (Figure 3). This preferential activation pattern likely reflects differences in receptor-binding affinity: previous studies demonstrated that PPARs are preferentially activated by PFCAs rather than PFSAs,^75,76^ supporting our findings and highlighting the interplay between PFAS chemistry and the activation of specific mechanistic pathways toward the PFAS-induced liver steatosis core mechanism. Thus, chemical structure introduces a second dimension of context dependency, shaping which system-specific mechanistic route preferentially activates the liver steatosis core of events.

Therefore, these findings suggest that PFAS-induced liver steatosis results from a conserved mechanistic core of events that can be engaged through multiple context-dependent routes, defined jointly by biological system properties, such as receptor expression profiles, and PFAS-specific determinants, including chemical structure, kinetics, and exposure dynamics. Sensitivity analysis across platforms, systems, and chemical classes confirmed the robustness of this core mechanism (Figure S10). Across all categories, the core mechanism remained consistently enriched, confirming that its identification is not dependent on any specific subset of the data. Importantly, the biological system-specific subnetworks further confirmed that each system independently activates the conserved core through distinct upstream entry points, strengthening the conclusion that context-dependent mechanistic branching is a robust feature of PFAS-induced liver steatosis rather than a consequence of data heterogeneity. At a broader level, this insight has important implications for PFAS hazard assessment. Commonly used strategies are insufficient to capture mechanistic similarity and toxicological potential supporting the need to move toward mechanistically informed, context-aware strategies that are better aligned with the principles of precision toxicology. Additionally, our comparative analysis of liver models of increasing complexity shows that simpler systems can capture the full multiscale mechanisms, including early molecular events that are attenuated in more complex models, providing practical guidance for test-system selection in predictive liver toxicity assessment. Moreover, identifying biological system–specific gene panels relevant for liver steatosis transforms this mechanistic insight into an actionable framework, enabling rapid screening of uncharacterized PFAS, supporting early prioritization, and reducing reliance on animal testing (Figure S11).

Finally, these patterns suggest that PFAS-induced steatosis may extend beyond the liver to the kidney. While PFAS preferentially accumulate in the liver,^76–78^ several compounds undergo efficient renal reabsorption, leading to renal accumulation.^79,80^ Epidemiological evidence links PFAS exposure, particularly PFOS, to renal dysfunction, often associated with inflammation and altered lipid metabolism while computational analyses similarly implicate lipid metabolism and PPAR signaling in renal outcomes.^81,82^ Given the high expression of PPARs in kidney epithelial cells,^73^ and dysregulation of lipid homeostasis as a shared feature, our findings raise the hypothesis that same steatosis mechanisms may also operate in the kidney, with the preferential mechanistic branch determined by the context. Although the lack of toxicogenomic data on kidney tissue prevents definitive confirmation, our contextualized AOP–based approach provides a mechanistically plausible model for a shared, multiorgan PFAS-induced steatosis mechanism.

### Investigation of PFAS KE–KE Network Reveals Time-Dependent PFAS Toxicity Pathways

Grouping enriched AOPs into human health hazard categories across exposure periods, we observed a clear impact of the temporal dimension on the number of activated AOPs (Figure 2). To capture these temporal dynamics, we constructed independent KE–KE networks for short-, intermediate-, and long-term exposures. Comparative analysis of these networks revealed temporal patterns of the PFAS-induced mechanisms.

We identified a time-dependent progression of KEs activated leading to liver steatosis, where increasing exposure duration correlated with the biological complexity of the activated events (Figure 4). This temporal pattern was observed for PFOS, PFOA, GenX, and PFNA, all of which exhibited complete mechanistic chains, from MIEs to the AO, across time scales. Interestingly, the whole mechanism was already deregulated at short and intermediate exposure periods, whereas at longer exposures, only the AO and its proximal KEs remained altered (Figure 4). This pattern indicates that early molecular perturbations induced by PFAS compounds evolve into apical and persistent phenotypes, reflecting the progression of the mechanism over time. However, the apparent loss of early molecular events over longer periods may be influenced by both biological and technical factors. Technically, longer duration studies are predominantly derived from lower-coverage transcriptomic platforms, which may limit the detection of early molecular signals. Biologically, molecular signals in transcriptional responses may be attenuated as higher level phenotypic effects dominate. In both cases, the observations support a dynamic, temporally structured mechanism progressing from initial molecular perturbations to stable AOs.

**Figure 4:**
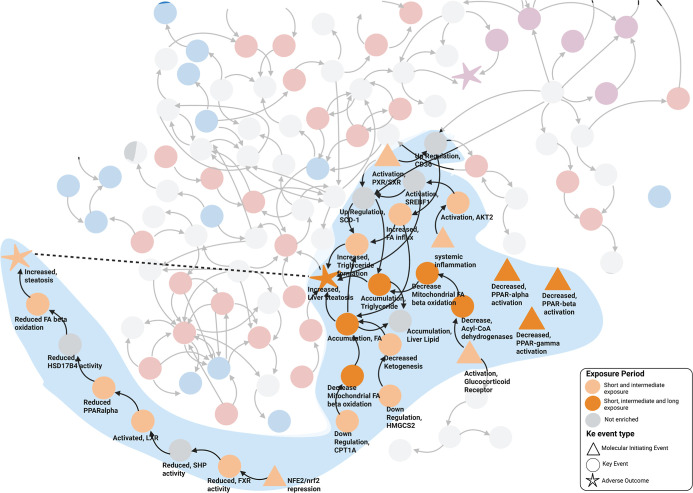
Liver steatosis mechanism progression in time. The figure represents the exposure period overlapping subnetwork of liver steatosis. Each node represents a KE, the shape refers to the type (MIE, KE or AO) while the color refers to the exposure period in which the KE occurs.

Beyond liver steatosis, AOP:69, whose mechanism was investigated above, also displayed a temporal pattern (Figure S5). Specifically, the number of PFAS activating this mechanism increased with the exposure duration, suggesting a cumulative or delayed MOA. At short exposure, KEs within AOP:69 were enriched by three PFAS, but this expanded to seven at intermediate and eight at long exposure (Figure S5). This temporal effect indicates that a mechanism initially associated with a few legacy PFAS may be broadly affected across the class, including emerging alternatives.^65^ Differences in PFAS uptake, transport, and intracellular accumulation may underlie the delayed activation of AOP:69 by many PFAS.^83^ Indeed, PFAS with slower uptake or stronger plasma protein binding may require prolonged exposure to reach intracellular concentrations sufficient to perturb cholesterol synthesis and steroidogenesis, while more permeable PFAS may elicit earlier responses.^83^ While this mechanistic hypothesis is plausible, confirming it would require a targeted investigation on PFAS-specific kinetics and endocrine activity, which are beyond the scope of this study.

Taken together, these analyses reveal that PFAS-induced mechanisms display clear temporal dependencies, likely reflecting compound-specific accumulation and delayed molecular activation.

Tissue-specific progression of shared MIEs drives PFAS systemic toxicity with immunotoxicity as a cross-context contributor.

Throughout this study, we show that both liver steatosis cascades of events and AOP:69 implicate PPAR activation as an MIE following PFAS exposure. Our findings, supported by existing literature, suggest that PPAR dysregulation may function as a shared initiating event linking otherwise distinct AOs.^65^ These suggest that tissue-specific AOs may originate from common molecular perturbations that evolve into tissue-specific effects through context-dependent signaling, providing a mechanistic explanation for the systemic toxicity observed across the PFAS classes.

To further explore this hypothesis, we scanned the KE–KE network to identify shared MIEs propagating toward multiple AOs. A notable example is activation of the aryl hydrocarbon receptor (AhR). AhR links PFAS exposure to lung fibrosis and breast cancer in macrophage-driven contexts through processes such as inflammation, collagen deposition, migration, and angiogenesis, while connecting to liver steatosis via crosstalk with NR pathways, in liver-related systems (Figure 5). These observations are supported by epidemiological evidence linking elevated serum PFAS levels to increased breast cancer risk and by reported associations between PFOA or PFNA exposure and chronic obstructive pulmonary disease,^84–86^ as well as molecular evidence of TGFβ/SMAD signaling pathway disruption,^87^ further supporting the biological plausibility of these mechanisms. AhR, a ligand-dependent transcription factor known to interact with PFAS,^88^ is broadly expressed across tissues and engages in extensive crosstalk with tissue-specific regulatory proteins^89^ triggering multiple downstream responses. Thus, the observation that PFAS–AhR interactions can contribute to multiple outcomes is therefore mechanistically consistent.

**Figure 5:**
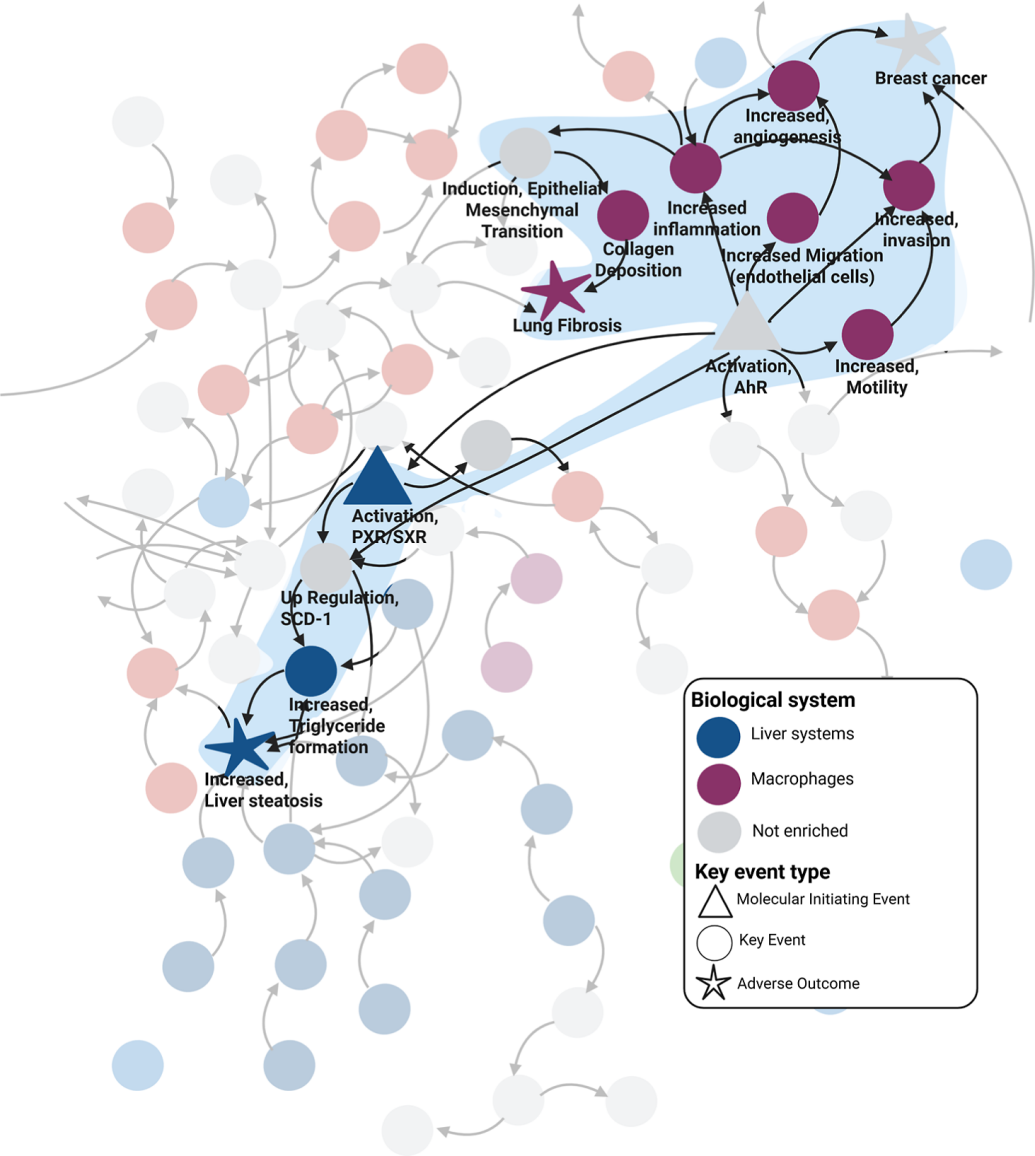
Shared PFAS molecular events progressing toward organ-specific adverse outcomes. This subnetwork illustrates the set of KEs representing early molecular perturbations shared across PFAS exposures, and how these events diverge into distinct organ-specific AOs depending on the biological context. Node shape indicates KE type, while node color denotes the biological system in which the KE is enriched. Edges represent KERs.

Importantly, our network analysis reveals inflammation as a central branching point through which shared molecular perturbations diverge into organ-specific toxicological end points. Immunotoxicity emerged as an activated end point (Figure 2B), with immunotoxicity-related AOPs converging on hyperinflammation (e.g., AOP:392 and AOP:406). Thus, PFAS exposure consistently modulates immune function through pro-inflammatory signaling, supported by existing literature and by the cytokine responses observed in PFAS-exposed macrophages generated in this study (Figure S10).^90^ Indeed, macrophage exposure to seven PFAS elicited dose-dependent pro-inflammatory responses for nearly all compounds tested, suggesting that immune activation may represent a mechanism that amplifies downstream organ-specific chains of events. These findings suggest that immunotoxicity should not be interpreted as an isolated toxicological end point but rather as a mechanistic process that facilitates the development of multiple organ-specific AOs. Its toxicological relevance is time-dependent: while acute inflammatory responses may resolve following stressor removal, chronic PFAS exposure, more reflective of real-world scenarios, may promote persistent inflammation, a well-established risk factor for fibrosis, carcinogenesis, and cardiometabolic disease.^91^

This hypothesis is reinforced by the activation of cardiovascular- and lung-related AOPs, despite limited representation of cardiac and pulmonary systems in the collection. These signals likely reflect systemic consequences of PFAS-induced lipid dysregulation and chronic inflammation, mechanisms known to contribute to cardiometabolic dysfunction and fibrotic lung disease in susceptible biological contexts.^92^ At the same time, although our results suggest mechanistic links between PFAS-induced immunomodulation and these outcomes, targeted studies using tissue-specific models are needed to characterize context-dependent responses and support the precision-level interpretation of PFAS toxicity.

### Implications for PFAS Safety Assessment

Current chemical safety assessment approaches struggle to address the thousands of chemicals in use and fail to account for toxicity emerging from interactions between multiple dimensions. The PFAS class exemplifies these limitations. By integrating a curated PFAS toxicogenomic collection with an AOP-based, context-aware framework, this study lays the foundation for a shift toward precision toxicology approaches. In this approach, AOs are understood as context-dependent pathways shaped by the biological system under study, without operationalizing it as a framework for individualized risk prediction, although it may inform potency ranking through dose-dependent analyses and support, without directly providing regulatory classification. Collectively, these findings frame PFAS systemic toxicity as originating from shared molecular perturbations that propagate in a context-dependent manner toward organ-specific outcomes rather than arising from inherently organ-specific mechanisms. The generation of toxicogenomic data in an immune context reveals inflammatory processes as contributors that amplify downstream toxicity across tissues, positioning immunomodulation not as an end point but as a recurrent mechanistic contributor to PFAS-associated adverse effects. In this context, the emerging mechanistic signals in underrepresented compartments, such as the kidney, and for less characterized PFAS illustrate the ability of our inherently inferential framework to prioritize relevant mechanisms for targeted, system-specific experimental validation. Despite these gaps, the curated PFAS toxicogenomic collection developed in this work provides an extensible resource for future data integration and refinement of PFAS mechanistic understanding. By openly providing the high-quality inclusion criteria, harmonization strategies, and analytical code, this resource can be readily expanded with future data sets or applied to other chemical classes, facilitating broader adoption of context-aware, mechanistically grounded toxicological assessments.

These mechanistic insights also have direct implications for safe and sustainable PFAS design strategies. Contrary to the assumptions underlying current substitution approaches, emerging PFASs are not intrinsically less hazardous than their legacy counterparts. Both experimental toxicity data and docking analyses indicate that legacy and emerging PFAS exhibit comparable binding affinities toward NRs that initiate convergent mechanisms, leading to liver steatosis. The primary difference lies in kinetics, with emerging PFASs often producing delayed effects, likely driven by differences in cellular uptake rather than reduced biological activity. Together, these observations highlight the limitations of design strategies that prioritize structural modification without mechanistic validation and emphasize the need to apply precision toxicology frameworks in the context of chemical design and regulatory decision making.

## Supplementary Material









## Notes

Detailed information on the public available entries included in the PFAS toxicogenomic collection (XLSX)
Detailed descriptors of the 388 exposure studies coming from the included entries (XLSX)
Reasons and number of studies excluded from the collection, counts of exposure studies by system × platform × PFAS × duration, PFAS subgrouping, distribution of exposure studies grouped by biological system and PFAS compound, exposure duration aggregation scheme, characterization of exposure studies based on exposure duration, sensitivity analysis of alternative thresholds for AOP fingerprint selection, refined causal mechanism of AOP:69, PFAS-induced liver steatosis subnetwork annotated by PFAS compound and biological system, JI similarity among liver steatosis related KE and binding affinity between PFAS included in the studies and selected nuclear receptors (PDF)
Information on data generation, dose selection, exposure, RNA extraction and sequencing, and cytokine secretion; detailed information on RNA seq, TempO-seq, and Microarray preprocessing workflow; effect on macrophages’ viability of PFAS congeners, dose-dependent effects of PFAS on cytokine secretion and the derivation of a panel of genes relevant for PFAS-induced liver steatosis grouped for biological system relevance (PDF)
