# Supplementary material for: AOP-Based Analysis of Curated Transcriptomic Data Reveals a Context-Dependent Core Mechanism of PFAS-Induced Liver Steatosis
Source: Environ Sci Technol. 2026 Jul 30;60(31):21495–509. doi: 10.1021/acs.est.6c02707 (PMC13472045; doi:10.1021/acs.est.6c02707)
Supplement: Supplementary file 1 [file es6c02707_si_003.pdf]

Supporting information III

AOP-based Analysis of Curated Transcriptomic Data Reveals a Context-dependent Core Mechanism of PFAS-induced Liver Steatosis

*Nicoletta D'Alessandro, Luca Mannino, Giusy del Giudice, Laura Ylä-Outinen, Noora Perho, Laura Aliisa Saarimäki, Emanuele Di Lieto, Zeyad Al-Abdulraheem, Marcella Torres Maia, Simo Inkala, Lorenzo Campini, Lena Möbus, Jack Morikka, Antonio Federico, Angela Serra, Dario Greco**

Supplementary figures and tables

Table S1. Table containing the number of entries discarded from the collection for each of the established inclusion criteria

Reason to discard	Number of unique entries
Incomplete metadata	7
Raw data unavailable	1
Marginally represented platform	2
Lack of control condition	1
Lack of replicates	1
Total dataset discarded	12

Table S2. Table summarizing the PFAS toxicogenomic collection characteristics, counting the exposure studies per platform, biological system, compound and exposure duration

Platform	Biological system	Compound	Exposure duration	Counts
DNA Microarray	liver	PFNA	Long-term exposure	2
DNA Microarray	liver	PFOS	Long-term exposure	1
DNA Microarray	fetal liver	PFOA	Intermediate-term exposure	4
DNA Microarray	fetal liver	PFOS	Intermediate-term exposure	2
DNA Microarray	fetal lung	PFOA	Intermediate-term exposure	2
DNA Microarray	fetal lung	PFOS	Intermediate-term exposure	2
DNA Microarray	liver	PFHxS	Intermediate-term exposure	2
DNA Microarray	liver	PFNA	Intermediate-term exposure	2
DNA Microarray	liver	PFOS	Intermediate-term exposure	2
DNA Microarray	spleen	PFOS	Intermediate-term exposure	1
DNA Microarray	whole organism	PFBA	Short-term exposure	2
RNA sequencing	liver	HFPO-DA	Long-term exposure	1
RNA sequencing	liver	PFOA	Long-term exposure	2
RNA sequencing	whole organism	4:2 Fts	Short-term exposure	1
RNA sequencing	whole organism	FBSA	Short-term exposure	1
RNA sequencing	whole organism	PFBS	Short-term exposure	1
RNA sequencing	whole organism	PFPeA	Short-term exposure	1
RNA sequencing	primary hepatocytes	HFPO-DA	Intermediate-term exposure	16
RNA sequencing	primary hepatocytes	HFPO-DA	Short-term exposure	32

RNA sequencing	liver	PFESA-BP2	Intermediate-term exposure	4
RNA sequencing	whole organism	PFHxS	Intermediate-term exposure	6
RNA sequencing	liver	PFOA	Intermediate-term exposure	9
TempO-seq	human liver spheroids	4:2 Fts	Long-term exposure	4
TempO-seq	human liver spheroids	5:3 acid	Long-term exposure	4
TempO-seq	human liver spheroids	5:3 acid	Short-term exposure	1
TempO-seq	human liver spheroids	6:2 FtOH	Long-term exposure	4
TempO-seq	human liver spheroids	6:2 FtOH	Short-term exposure	4
TempO-seq	human liver spheroids	6:2 Fts	Long-term exposure	4
TempO-seq	human liver spheroids	6:2 Fts	Short-term exposure	3
TempO-seq	human liver spheroids	6:2 monoPAP	Long-term exposure	3
TempO-seq	human liver spheroids	6:2 monoPAP	Short-term exposure	1
TempO-seq	human liver spheroids	8:2 FtOH	Long-term exposure	4
TempO-seq	human liver spheroids	8:2 FtOH	Short-term exposure	3
TempO-seq	human liver spheroids	8:2 Fts	Long-term exposure	4
TempO-seq	human liver spheroids	8:2 Fts	Short-term exposure	4
TempO-seq	human liver spheroids	8:2 monoPAP	Long-term exposure	4
TempO-seq	human liver spheroids	PFBA	Long-term exposure	4
TempO-seq	human liver spheroids	PFBA	Short-term exposure	4
TempO-seq	human liver spheroids	PFBS	Long-term exposure	20
TempO-seq	human liver spheroids	PFBS	Short-term exposure	7

TempO-seq	human liver spheroids	PFDA	Long-term exposure	2
TempO-seq	human liver spheroids	PFDA	Short-term exposure	1
TempO-seq	human liver spheroids	PFDS	Long-term exposure	20
TempO-seq	human liver spheroids	PFDS	Short-term exposure	6
TempO-seq	human liver spheroids	PFHpA	Long-term exposure	4
TempO-seq	human liver spheroids	PFHpS	Long-term exposure	4
TempO-seq	human liver spheroids	PFHpS	Short-term exposure	2
TempO-seq	human liver spheroids	PFHxA	Long-term exposure	4
TempO-seq	human liver spheroids	PFHxA	Short-term exposure	4
TempO-seq	human liver spheroids	PFHxS	Long-term exposure	3
TempO-seq	human liver spheroids	PFHxS	Short-term exposure	4
TempO-seq	human liver spheroids	PFNA	Long-term exposure	2
TempO-seq	human liver spheroids	PFNA	Short-term exposure	3
TempO-seq)	human liver spheroids	PFOA	Long-term exposure	20
TempO-seq	human liver spheroids	PFOA	Short-term exposure	7
TempO-seq	human liver spheroids	PFOS	Long-term exposure	20
TempO-seq	human liver spheroids	PFOS	Short-term exposure	7
TempO-seq	human liver spheroids	PFOSA	Long-term exposure	4
TempO-seq	human liver spheroids	PFOSA	Short-term exposure	3
TempO-seq	human liver spheroids	PFPeA	Long-term exposure	4
TempO-seq	human liver spheroids	PFPeA	Short-term exposure	3

TempO-seq	human liver spheroids	PFTeDA	Long-term exposure	6
TempO-seq	human liver spheroids	PFTeDA	Short-term exposure	6
TempO-seq	human liver spheroids	PFUnA	Long-term exposure	2
TempO-seq	human liver spheroids	PFUnA	Short-term exposure	4
TempO-seq	liver	PFESA-BP2	Intermediate-term exposure	4
RNA sequencing	macrophages	8:2 cl-PFESA	Intermediate-term exposure	6
RNA sequencing	macrophages	8:2 cl-PFESA	Short-term exposure	3
RNA sequencing	macrophages	Branched AD ONA	Intermediate-term exposure	6
RNA sequencing	macrophages	Branched AD ONA	Short-term exposure	3
RNA sequencing	macrophages	PFESA-BP1	Intermediate-term exposure	6
RNA sequencing	macrophages	PFESA-BP1	Short-term exposure	3
RNA sequencing	macrophages	PFESA-BP2	Intermediate-term exposure	5
RNA sequencing	macrophages	PFESA-BP2	Short-term exposure	2
RNA sequencing	macrophages	PFHpS	Intermediate-term exposure	6
RNA sequencing	macrophages	PFHpS	Short-term exposure	3
RNA sequencing	macrophages	PFOA	Intermediate-term exposure	6
RNA sequencing	macrophages	PFOA	Short-term exposure	3
RNA sequencing	macrophages	PFOS	Intermediate-term exposure	6
RNA sequencing	macrophages	PFOS	Short-term exposure	3

Table S3. Table reporting the current AOP status of the 42 unique AOPs enriched by the curated PFAS collection

AOP_ID	AOP_name	AOP_status
**Aop:1**	Uncharacterized liver damage leading to hepatocellular carcinoma	none
**Aop:104**	Altered ion channel activity leading impaired heart function	none
**Aop:115**	Epithelial cytotoxicity leading to forestomach tumors (in mouse and rat)	none
**Aop:116**	Cytotoxicity leading to renal tubular adenomas and carcinomas (in male rat)	none
**Aop:117**	Androgen receptor activation leading to hepatocellular adenomas and carcinomas (in mouse an d rat)	Under Development
**Aop:118**	Chronic cytotoxicity leading to hepatocellular adenomas and carcinomas (in mouse and rat)	none
**Aop:139**	Alkylation of DNA leading to cancer 1	none
**Aop:166**	PPARalpha activation leading to pancreatic acinar tumors in the rat and mouse	none
**Aop:206**	Peroxisome proliferator-activated receptors gamma inactivation leading to lung fibrosis	Under Development
**Aop:213**	Inhibition of fatty acid beta oxidation leading to nonalcoholic steatohepatitis (NASH)	none
**Aop:263**	Uncoupling of oxidative phosphotylation leading to reduced growth (1)	WPHA/WNT Endorsed
**Aop:274**	Histone deacetylase inhibition leads to impeded craniofacial development	none
**Aop:279**	Microtubule interacting drugs lead to peripheral neuropathy	none
**Aop:282**	Reactive oxygen species generated from photoreactive chemicals leading to phototoxic reaction s	Under Review
**Aop:285**	Inhibition of N-linked glycosylation leads to liver injury	none
**Aop:288**	Inhibition of 17alpha-hydrolase/C 10,20- lyase (Cyp17A1) activity leads to birth reproductive defects (cryptorchidism) in male (mammals)	none
**Aop:306**	Androgen receptor (AR) antagonism leading to short anogenital distance (AGD) in male (mammal ian) offspring	Under Review
**Aop:318**	Glucocorticoid Receptor activation leading to hepatic steatosis	none
**Aop:319**	ACE2 inhibition leading to lung fibrosis	Under Development
**Aop:344**	Androgen receptor (AR) antagonism leading to nipple retention (NR) in male (mammalian) offspri ng	Under Development
**Aop:345**	Androgen receptor (AR) antagonism leading to decreased fertility in females	Under Development
**Aop:36**	Peroxisomal Fatty Acid Beta-Oxidation Inhibition Leading to Steatosis	none
**Aop:374**	Binding of Sars-CoV- 2 spike protein to ACE 2 receptors expressed on brain cells (neuronal and non- neuronal) leads to neuroinflammation resulting in encephalitis	Under Development
**Aop:382**	Angiotensin II type 1 receptor (AT1R) agonism leading to lung fibrosis	Under Development
**Aop:385**	ACE2 dysregulation leading to microvascular disfunction	none
**Aop:392**	Decreased fibrinolysis and activated bradykinin system leading to hyperinflammation	Under Development
**Aop:394**	Sars-cov-2 infection leading to impaired olfactory function (anosmia)	Under Development
**Aop:400**	Increased (ectopic) concentration of all- trans retinoic acid (ATRA) if fetal testis leading to reduced sperm count, males	none
**Aop:406**	SARS-CoV-2 infection leading to hyperinflammation	none
**Aop:420**	Aryl hydrocarbon receptor activation leading to lung cancer through sustained NRF2 toxicity path way	none
**Aop:428**	Binding of S-protein to ACE2 in enterocytes induces ACE2 dysregulation leading to gut dysbiosis	Under Development
**Aop:433**	hERG inhibition leading to cardiac toxicity	none
**Aop:439**	Activation of the AhR leading to breast cancer	Under Review

**Aop:60**	NR1I2 (Pregnane X Receptor, PXR) activation leading to hepatic steatosis	none
**Aop:62**	AKT2 activation leading to hepatic steatosis	none
**Aop:69**	Modulation of Adult Leydig Cell Function Subsequent to Decreased Cholesterol Synthesis or Tra nsport in the Adult Leydig Cell	none
**Aop:106**	Chemical binding to tubulin in oocytes leading to aneuploid offspring	Under Review
**Aop:15**	Alkylation of DNA in male pre-meiotic germ cells leading to heritable mutations	WPHA/WNT Endorsed
**Aop:232**	NFE2/Nrf2 repression to steatosis	none
**Aop:284**	Binding of electrophilic chemicals to SH(thiol)-group of proteins and /or to seleno-proteins involved in protection against oxidative stress leads to chronic kidney disease	none
**Aop:296**	Oxidative DNA damage leading to chromosomal aberrations and mutations	WPHA/WNT Endorsed
**Aop:397**	Bulky DNA adducts leading to mutations	Under Development

Table S4. Subgroup classification of PFAS included in this study. The first column reports the abbreviated name of each PFAS, and the second column indicates its structural subgroup based on headgroup chemistry. Ether-PFAS were assigned to the same subgroup as their non-ether analogues when they shared the same headgroup.

Abbreviation	PFAS subgroup
PFOA	Perfluoroalkyl Carboxylic Acids - PFCAs
Branched ADONA	
PFBA	
5:3 FTCA	
PFHxA	
PFPeA	
HFPO-DA	
PFDA	
PFUnA	
PFNA	
PFTeDA	
PFHpA	
PFOS	Perfluoroalkyl Sulfonic Acids - PFSAs
PFHxS	
PFHpS	
PFBS	
PFDS	
8:2 Cl-PFESA	
PFESA-BP1	
PFESA-BP2	
4:2 FTS	Fluorotelomer Sulfonic Acids - FTSAs
6:2 FTS	
8:2 FTS	
6:2 FTOH	Fluorotelomer Alcohols - FTOHs
8:2 FTOH	
6:2 monoPAP	Polyfluoroalkyl Phosphoric Acid Mono-esters - monoPAPs
8:2 monoPAP	
FBSA	Perfluoroalkyl Sulfonamides - FASAs
PFOSA	

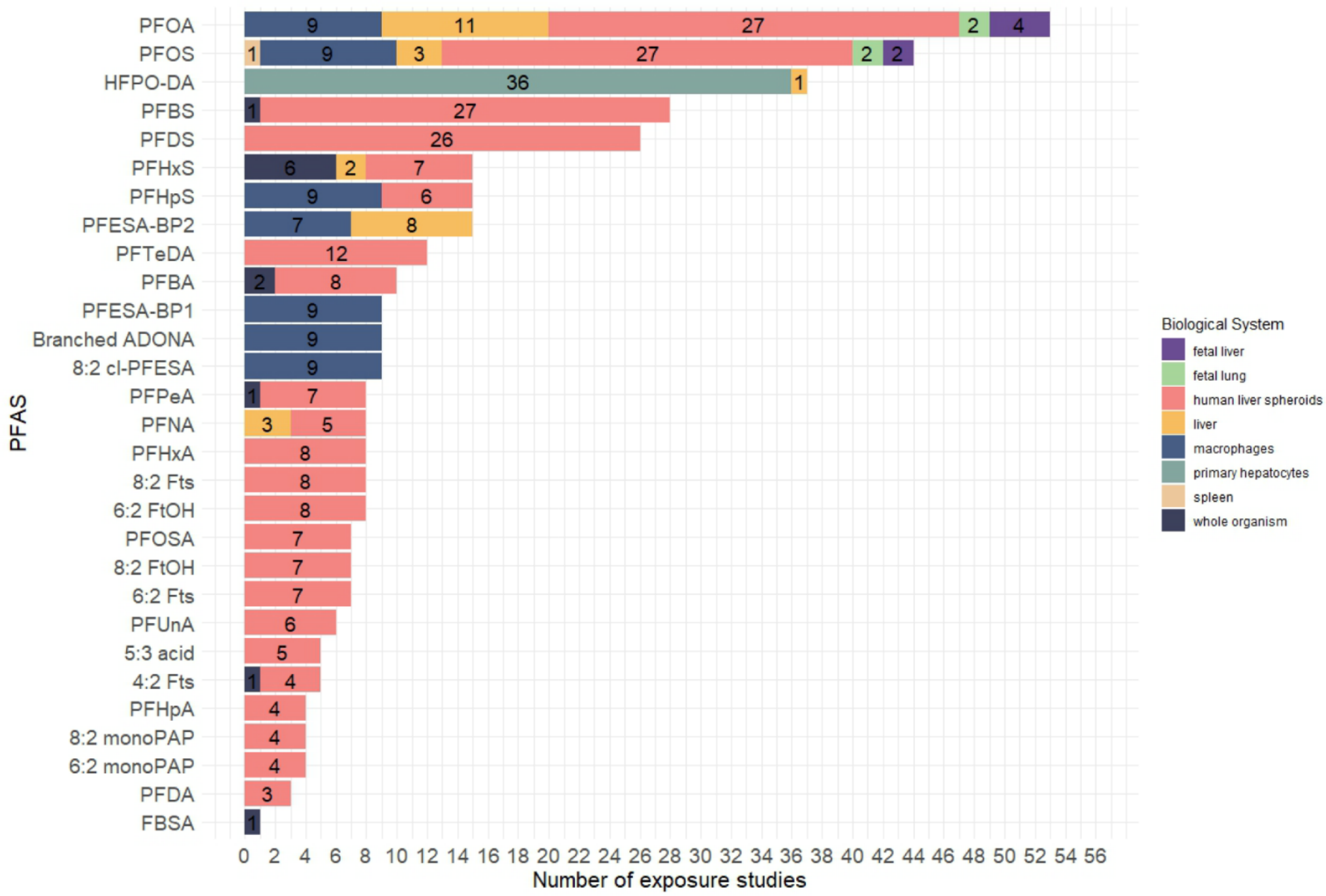

Figure S1. Distribution of exposure studies grouped by biological system and PFAS compound. The bar plot represents the number of exposure studies grouped by each PFAS (y axis) and biological system (colored bar)

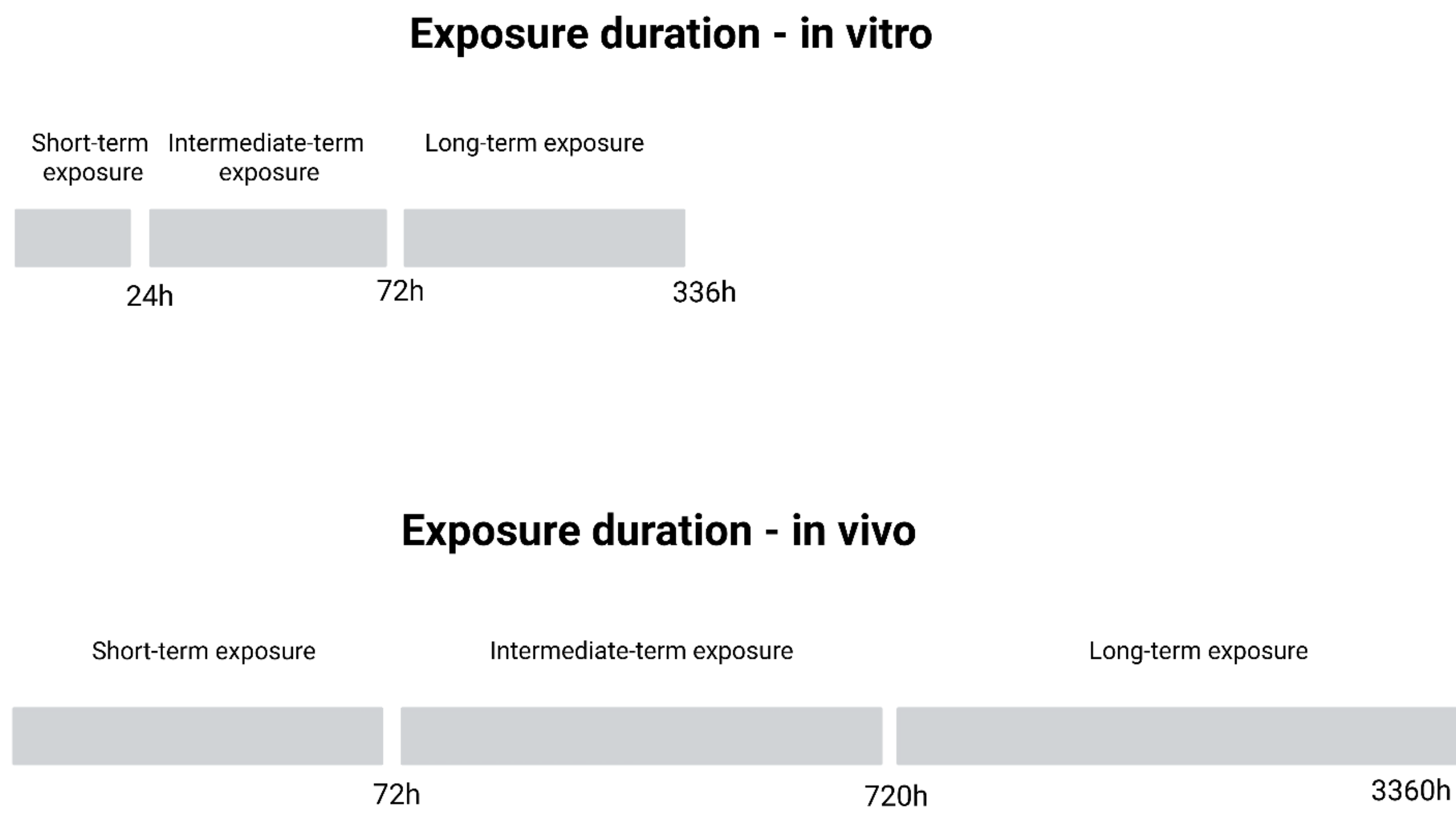

Figure S2. Classification of exposure duration of PFAS exposure based on exposure system. The different timelines for in-vitro and in-vivo systems are based on [4,33].

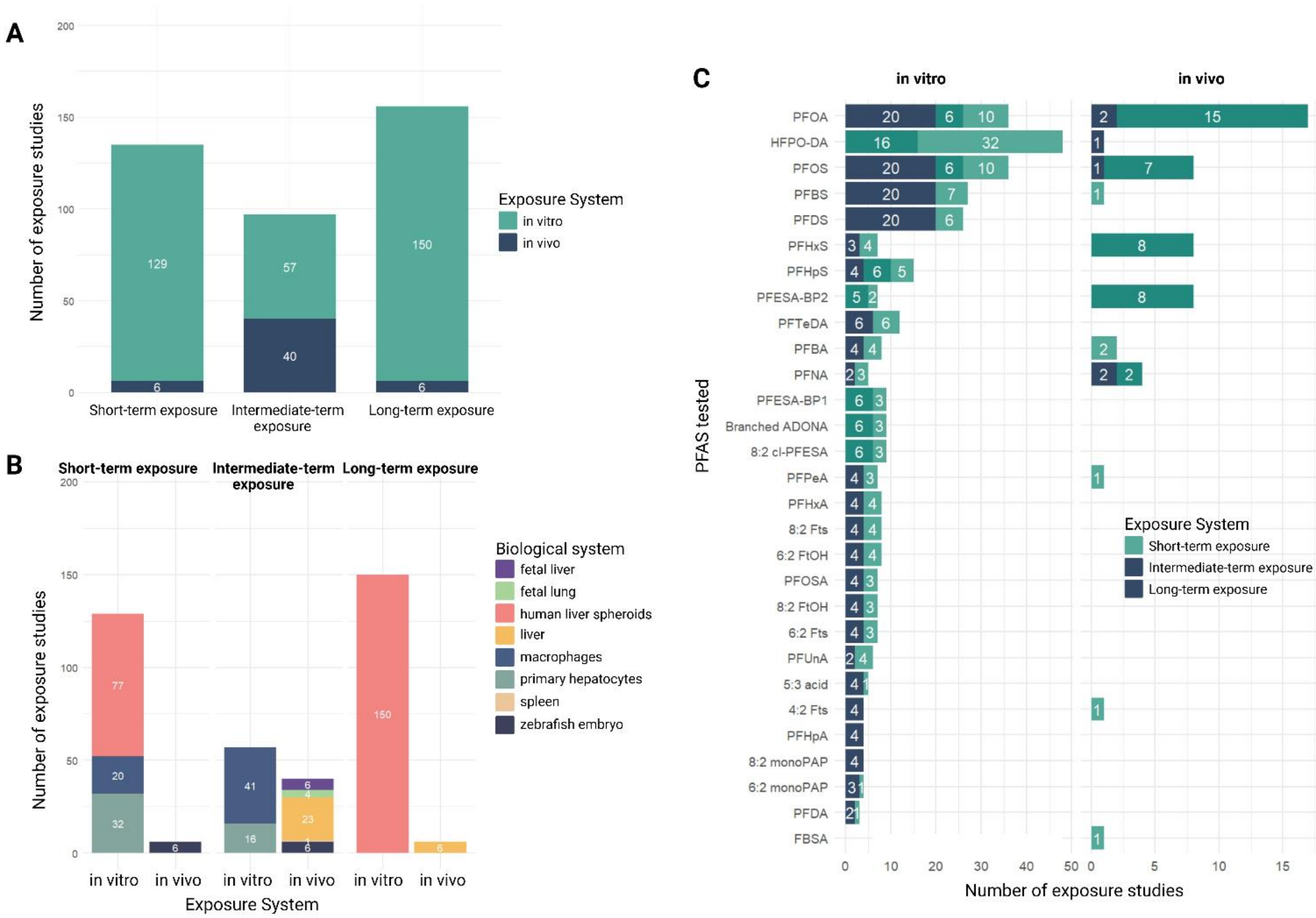

Figure S3. Characterization of exposure studies grouped by exposure periods. (A) The bar plot represents the distribution of exposure studies for each exposure periods colored by exposure system. (B) The bar plot represents the sample source distribution across exposure periods divided by exposure system. (C) the bar plot represents the distribution of exposure studies in which each PFAS has been tested across colored by exposure period and divided by exposure system. Each one of the PFAS present in the collection was tested in at least two different exposure periods, with legacy PFAS having data in all time ranges Similarly, all biological systems, with the exception of fetal lung and liver, were tested in at least two time points.

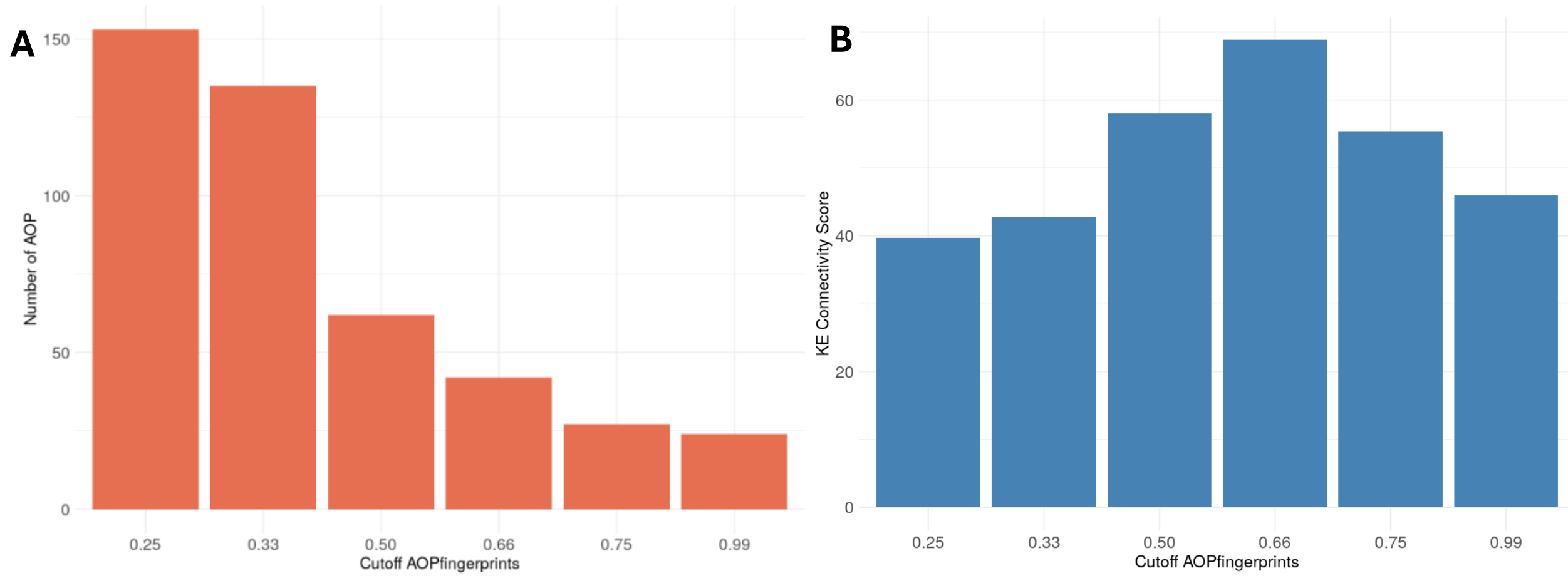

Figure S4. Sensitivity analysis of alternative thresholds for AOP fingerprint selection. (A) Number of AOPs retained under different thresholds based on the proportion of significantly enriched KEs within each AOP. (B) KE connectivity score calculated for each threshold, reflecting the proportion of connected versus isolated KEs within the reconstructed KE–KE network.

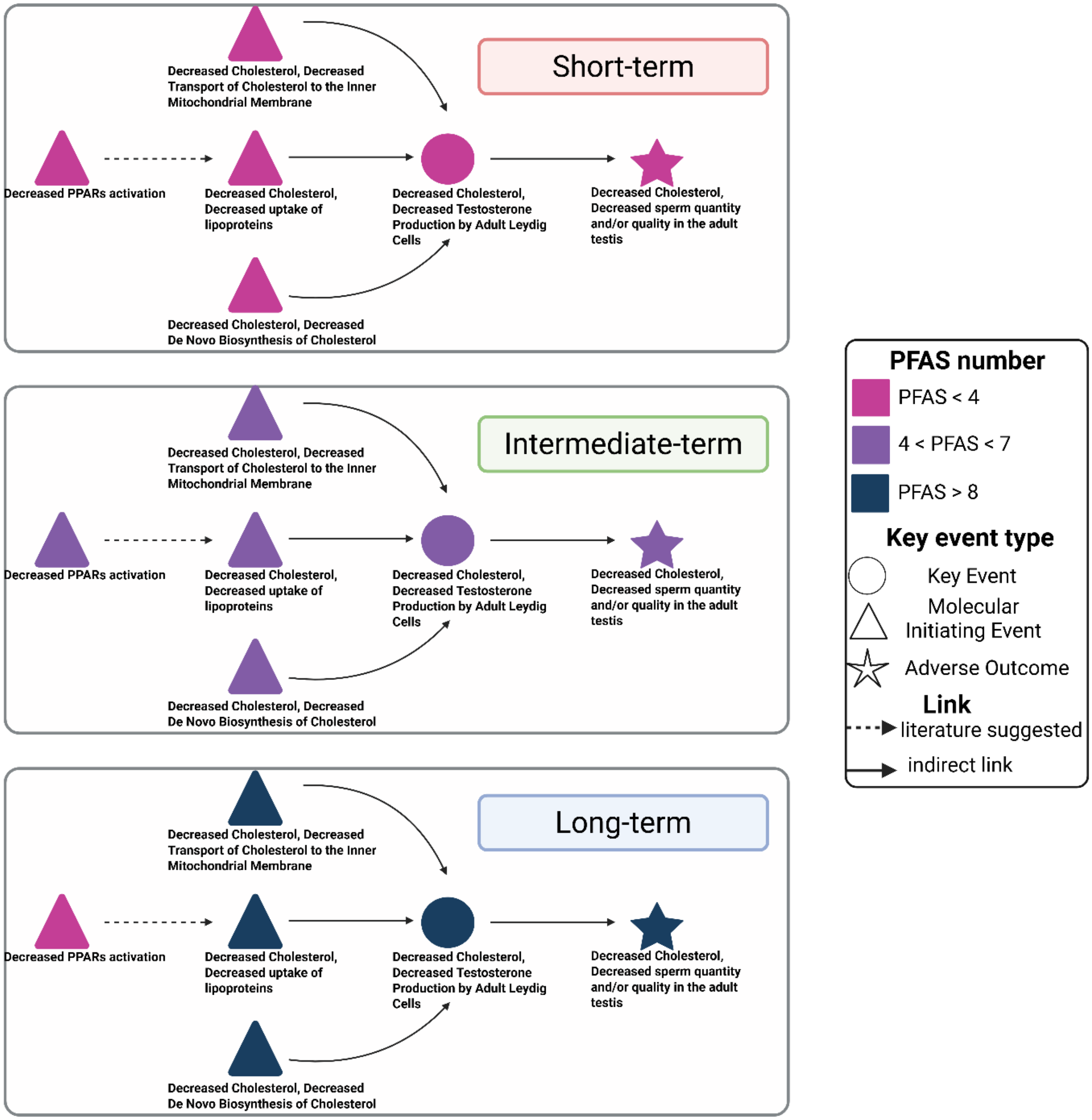

Figure S5. Graphical representation of the proposed causal mechanism of Aop:69 across exposure periods. Each rectangle represents an exposure period and contains the KEs enriched belonging to the AOP. Each node has a shape according to the type of event and is connected with an indirect link or a literature suggested link while the colors refer to the number of PFAS that alters each specific KE.

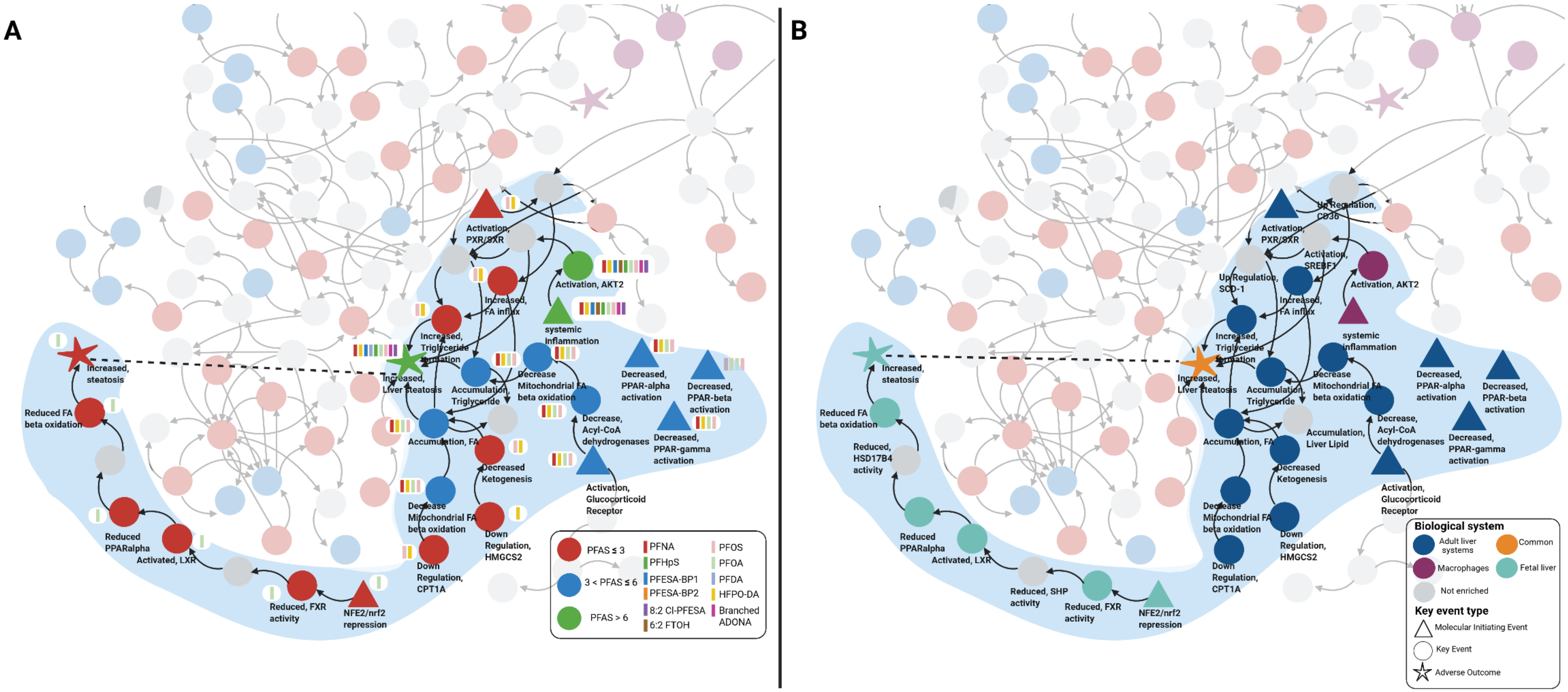

Figure S6. Context-dependent mechanistic subnetwork of PFAS-induced liver steatosis. Subnetwork illustrating the liver steatosis mechanism, with nodes representing key events and edges indicating their causal relationships. Node shape denotes Ke type. **(A)** Node color reflects the number of PFAS that significantly enriched each KE while colored bars indicate the specific PFAS contributing to that enrichment (one bar for each PFAS, color code in the legend). **(B)** Nodes color annotated according to the biological system in which each KE was activated.

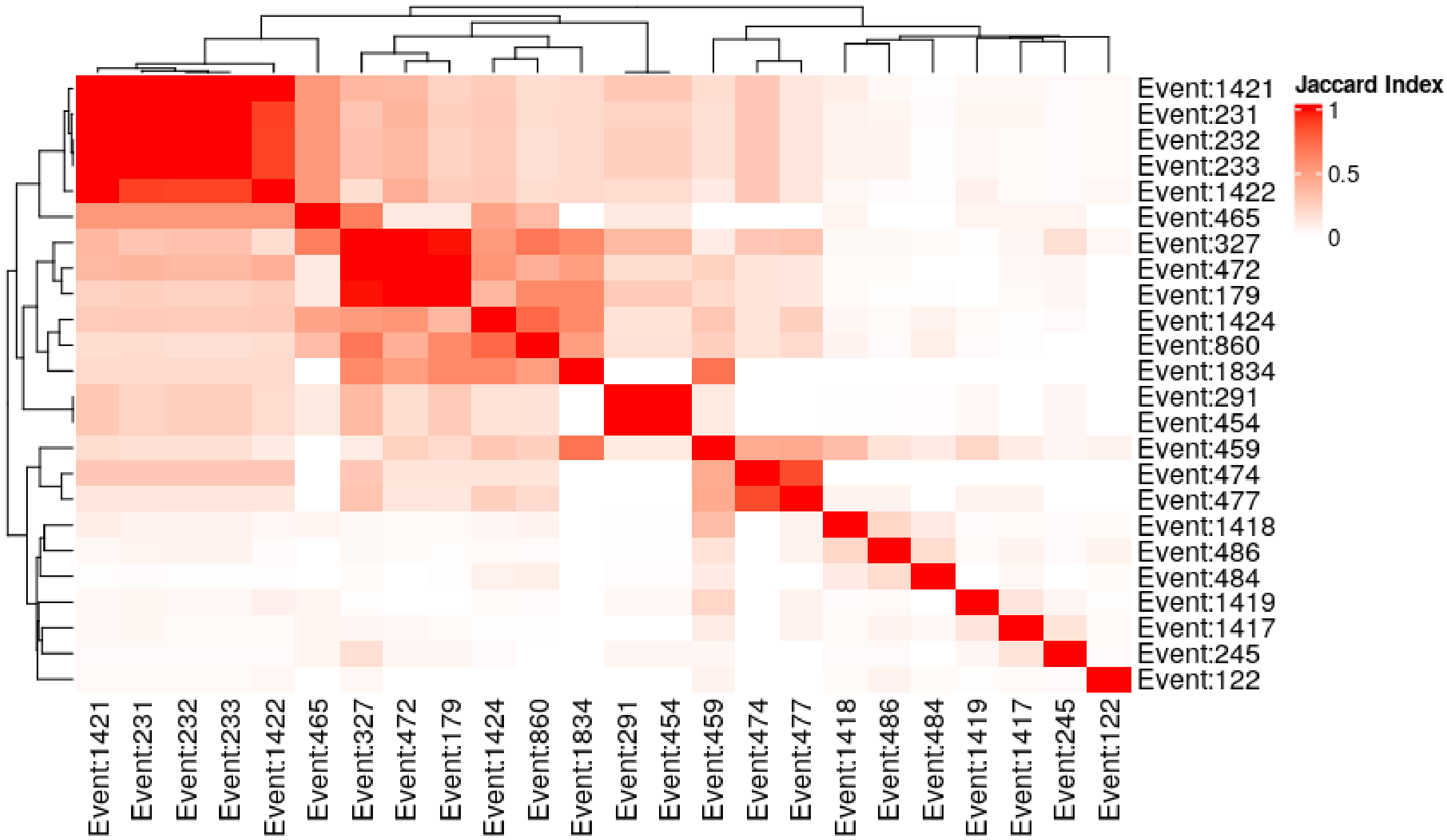

Figure S7. Jaccard index similarity among liver steatosis key events. The heatmap displays the gene set similarity between all pairs of liver steatosis key events, quantified using the Jaccard index normalized by gene set size. Colors range from white (no similarity, JI = 0) to red (highest similarity, JI = 1), with increasing intensity indicating greater overlap between key event–associated gene sets.

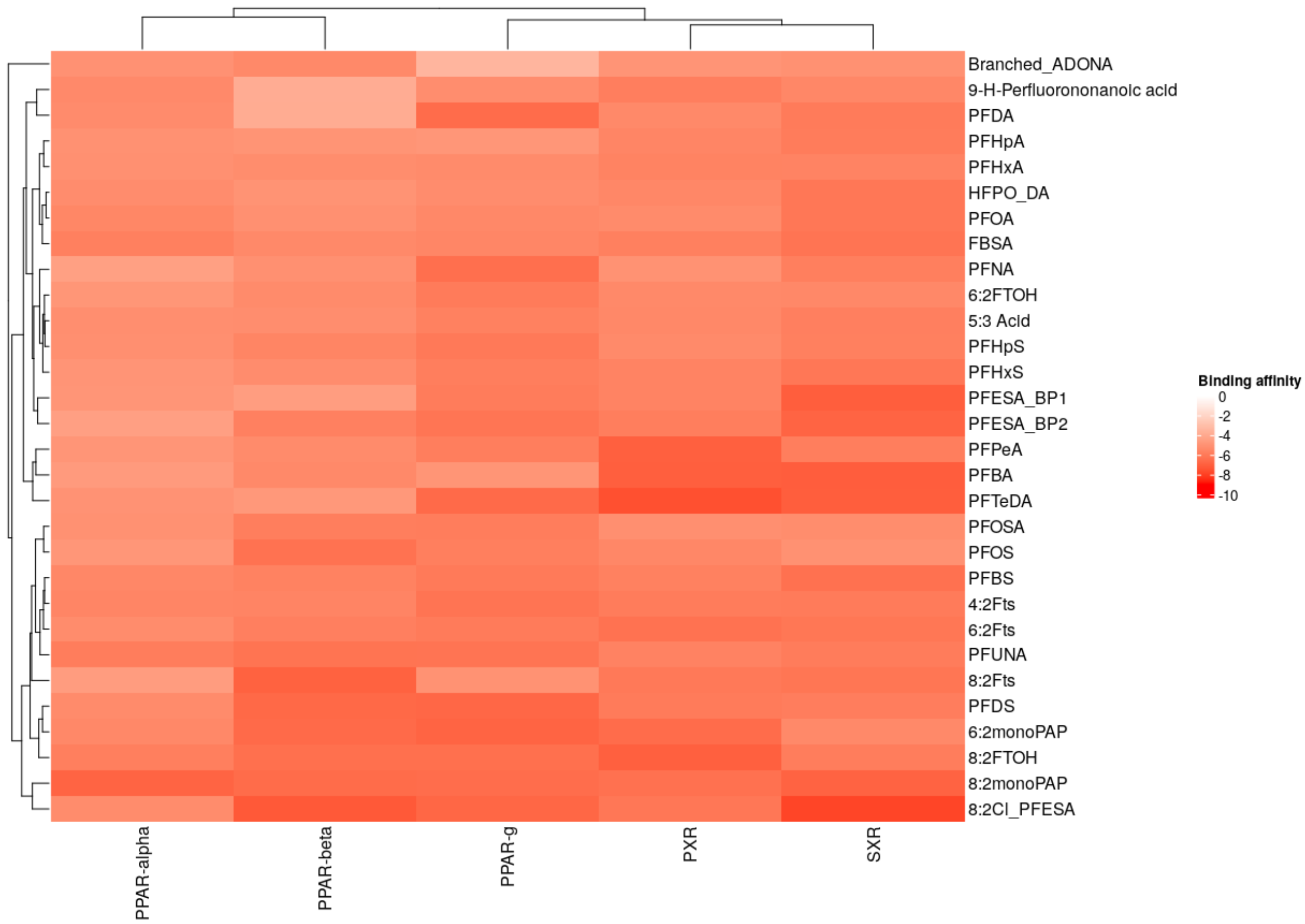

Figure S8. Binding affinity heatmap of PFAS–nuclear receptor interactions. Heatmap depicting the computed binding affinities between PFAS included in the toxicogenomic collection (y-axis) and selected nuclear receptors (x-axis). Color intensity reflects predicted binding strength, with increasing red intensity indicating higher binding affinity (lower docking scores).

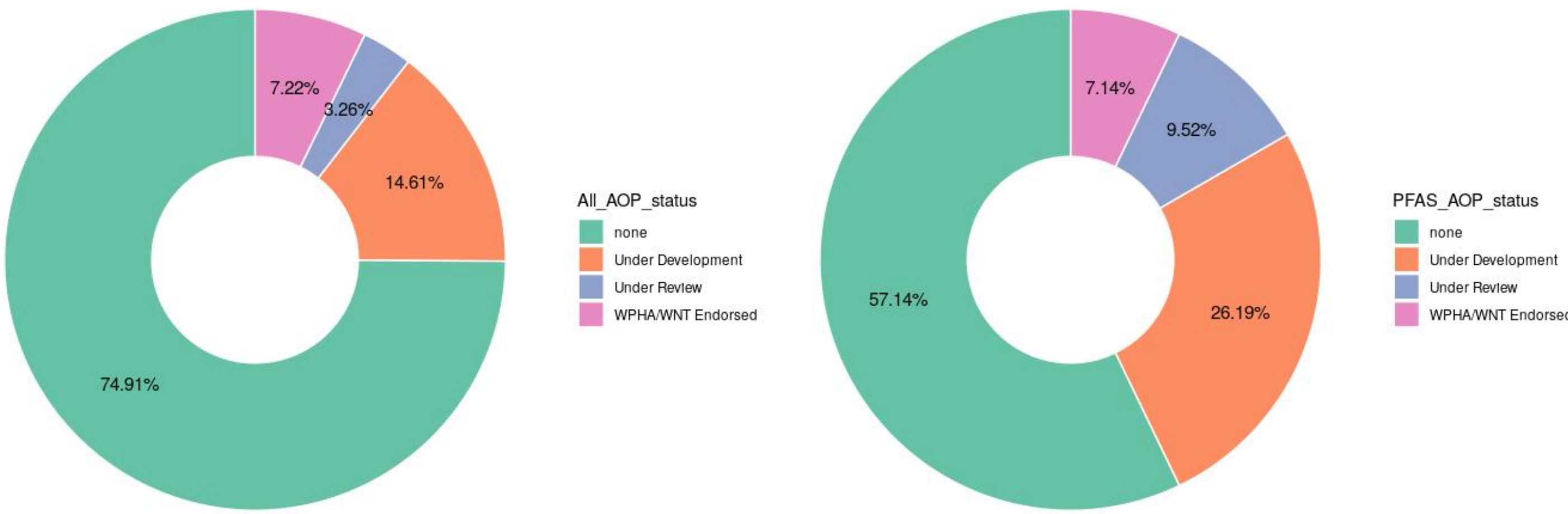

Figure S9. Current AOPs endorsement status. (Left) Donut chart representing the current AOP status of the whole AOP-wiki; (Right) Donut chart representing the current AOP status of the 42 unique AOPs enriched by PFAS curated collection.

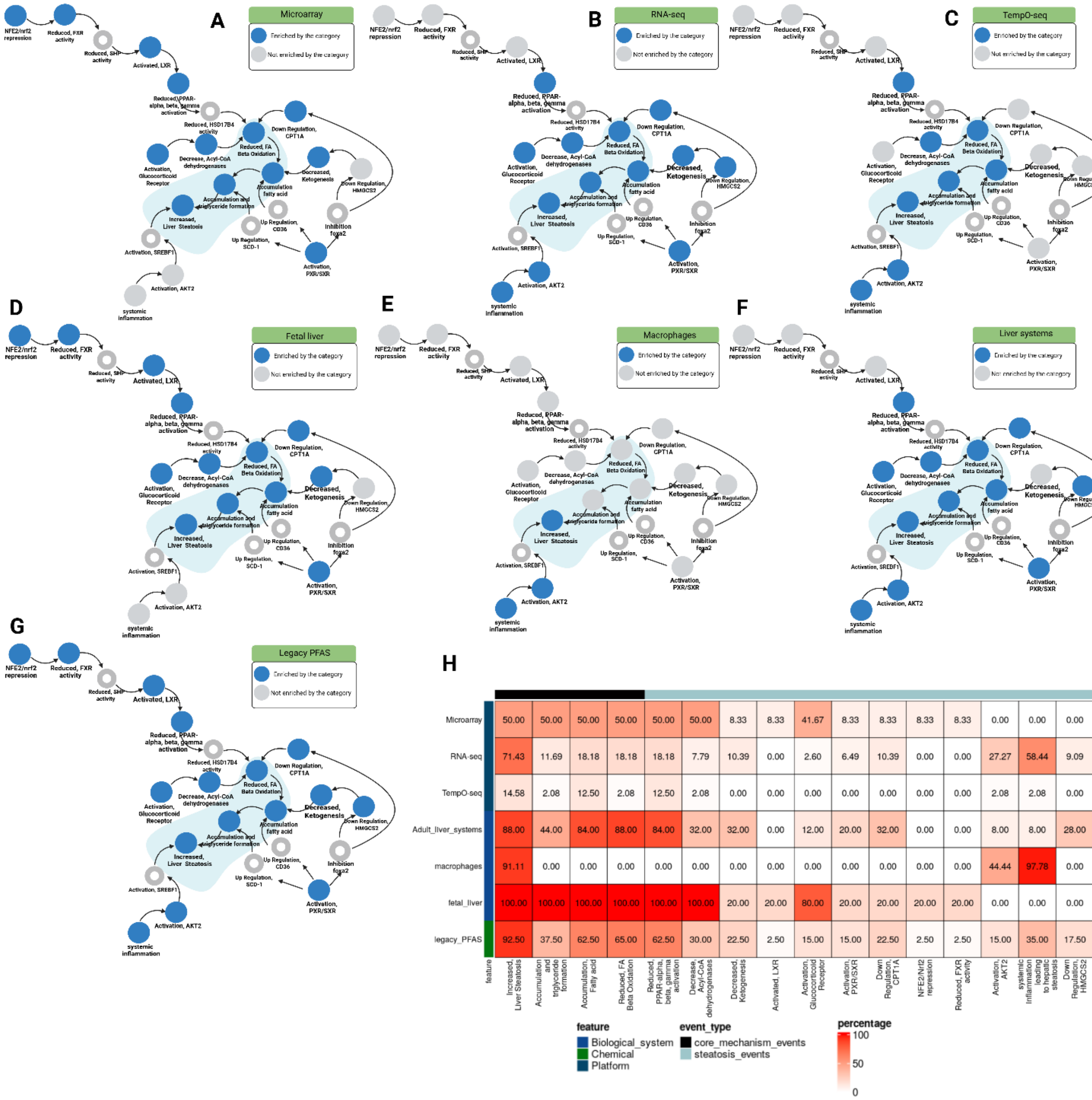

Figure S10. Sensitivity analysis of the PFAS-induced liver steatosis mechanism across dataset categories. Category-specific subnetworks of the PFAS-induced liver steatosis mechanism were generated by restricting the analysis to exposure studies belonging to each dataset category independently. Panels A–C show subnetworks derived from studies stratified by transcriptomic platform: microarray (A), RNA-seq (B), and TempO-seq (C). Panels D–F show subnetworks derived from studies stratified by biological system: fetal liver (D), macrophages (E), and adult liver systems (F). Panel G shows the subnetwork derived from legacy PFAS studies only. In all subnetworks, the blue cloud highlights the set of key events constituting the core mechanism of PFAS-induced liver steatosis. Panel H shows a heatmap reporting, for each dataset category (rows) and each key event of the full PFAS-induced liver steatosis mechanism (columns), the percentage of exposure studies within that category contributing to the enrichment of that event.
