# Supplementary material for: AOP-Based Analysis of Curated Transcriptomic Data Reveals a Context-Dependent Core Mechanism of PFAS-Induced Liver Steatosis
Source: Environ Sci Technol. 2026 Jul 30;60(31):21495–509. doi: 10.1021/acs.est.6c02707 (PMC13472045; doi:10.1021/acs.est.6c02707)
Supplement: Supplementary file 2 [file es6c02707_si_004.pdf]

Supporting information IV

AOP-based Analysis of Curated Transcriptomic Data Reveals a Context-dependent Core Mechanism of PFAS-induced Liver Steatosis

*Nicoletta D'Alessandro, Luca Mannino, Giusy del Giudice, Laura Ylä-Outinen, Noora Perho, Laura Aliisa Saarimäki, Emanuele Di Lieto, Zeyad Al-Abdulraheem, Marcella Torres Maia, Simo Inkala, Lorenzo Campini, Lena Möbus, Jack Morikka, Antonio Federico, Angela Serra, Dario Greco**

PFAS subtoxic concentrations determination based on cell viability assay and equipotent concentration exposure for RNA sequencing

THP-1 human monocytic cells (ATCC, #TIB-202) were cultured at 37 °C in a humidified incubator with 5% CO_2 in RPMI-1640 medium (ATCC, #30-2001) supplemented with 10% fetal bovine serum (FBS, Gibco, #A5256701). After thawing, cells were expanded for three passages before seeding in 384-well plates (Thermo Scientific, #142761) at a density of 1.05 × 10^5 cells/cm^2 (8,820 cell/well). To induce differentiation into macrophages, THP-1 were treated with 50 nM of phorbol 12-myristate 13-acetate (PMA, Sigma-Aldrich, #P1585) for 48 h. Following differentiation, the medium was replaced with exposure medium comprising RPMI-1640 containing 5% FBS, RealTime-Glo™ MT Cell Viability Assay reagent (Promega, #G9713), according to the manufacturer's instructions. Seven PFAS were tested in this study. Compound names, acronyms, CAS numbers, and suppliers are listed in Table 1. Stock solutions were freshly prepared in dimethyl sulfoxide (DMSO, Sigma-Aldrich, D2650) at 0.5 M on the day of exposure and diluted in exposure medium to achieve the desired final concentrations. The final DMSO concentration in all wells, including vehicle controls, did not exceed 0.1% (v/v). Following 48 h of differentiation, cells were exposed to a range of PFAS concentrations (0–1000 µM, 9 concentrations) for up to 72 h (Table S5). Each concentration was tested in four biological replicates and luminescence was measured after 24 h, 48 h, and 72 h of exposure using a Tecan Spark multimode plate reader.

Dose–response curves were generated for each PFAS and time point based on normalized luminescence values relative to vehicle controls. Concentration–response data were fitted using a non-linear regression model (four-parameter logistic fit) in GraphPad Prism. Equipotent concentrations were determined from 72-h exposure data, corresponding to the concentration inducing a comparable reduction in viability (Table S6). To characterize transcriptomic profile changes associated with PFAS exposure, the three sub-toxic equipotent concentrations identified for each PFAS were tested for RNA-sequencing analysis. The experimental workflow used for this experiment was equivalent to that of the viability assay, with the exception of cell culture scale. THP-1 cells were seeded in 12-well plates (Thermo scientific #150628) at the same density used for the viability experiments and differentiated with PMA under identical conditions. Following differentiation, cells were exposed to the three subtoxic concentrations of each PFAS for 24 h, 48 h, and 72 h in three biological replicates. At each timepoint, cells were harvested for total RNA extraction and subsequent sequencing and supernatant collected for cytokine assay.

Table S5. Detailed information about PFAS congeners tested in the study.

Abbreviation	Full name	Alternative names	CAS nro	Provider	cat nro
**PFOA**	Perfluorooctanoid acid		335-67-1	Sigma-Aldrich	#171468
**PFOS**	Perfluorooctanesulfonic acid		1763-23-1	Sigma-Aldrich	#77282
**PFHpS**	Perfluoroheptanesulfonic acid		375-92-8	APOLLO Scientific	PC53170
**PFESA BP2**	7H-Perfluoro-4-methyl-3,6-dioxaoctanesulfonic acid	Nafion by-product 2	749836-20-2	APOLLO Scientific	PC28311
**PFESA BP1**	1-(Trifluorovinyloxy)-2-(2-sulfotetrafluoroethoxy) hexafluoropropane	Nafion by-product 1	29311-67-9	APOLLO Scientific	PC28310
**Branched ADONA**	Perfluoro-2,5-dimethyl-3,6-dioxaheptanoic acid	DOPFLCA	2479-73-4	APOLLO Scientific	PC99180
**8:2 Cl-PFESA**	Potassium 2-[(8-chloro-1,1,2,2,3,3,4,4,5,5,6,6,7,7,8,8-hexadecafluorooctyl)oxy]-1,1,2,2-tetrafluoroethanesulfonate	8:2 Cl-PFAES	83329-89-9		

Table S6. Equipotent concentrations determined for each PFAS congeners for transcriptomics analysis.

	**vehicle control**	**low**	**middle**	**high**
**PFOA**	0,16% DMSO	100 µM	250 µM	400 µM
**PFOS**	0,2%DMSO	300 µM	400 µM	500 µM
**PFHpS**	0,28% DMSO	400 µM	550 µM	700 µM
**PFESA-BP2**	0,06% DMSO	20 µM	85 µM	150 µM
**PFESA-BP1**	0,06% DMSO	50 µM	100 µM	150 µM
**Branched ADONA**	0,32% DMSO	200 µM	500 µM	800 µM
**8:2 Cl-PFESA**	0,08% DMSO	100 µM	150 µM	200 µM

Cytokine secretion assay, RNA extraction, library preparation and sequencing

The cytokine secretion assay was performed on supernatant collected after 24h, 48 and 72h of exposure using V-plex proinflammatory panel 1 (MSD, #K15049D) and Meso QuickPlex SQ120 device (MSD) following the manufacturer's instructions. Ten cytokines were included in the plex: IFN-γ, IL-1β, IL-2, IL-4, IL-6, IL-8, IL-10, IL-12p70, IL-13, TNF-α.

Total RNA was extracted from the same samples using the Qiagen RNeasy kit according to the instruction of the provider (Qiagen #74106) complemented with DNase 1 (Thermo Scientific, EN0521) treatment. Library preparation and RNA sequencing was performed by Novogene according to their standard protocols. Prior to samples sending their quality was determined using Bioanalyzer 2100 (Agilent Technologies, USA) and only the samples with a RIN value higher than 5 were sent for sequencing. Libraries were sequenced using the Illumina Novaseq X platform, according to effective library concentrations and data amounts using the paired-end 150 strategy (PE150).

Data analysis of cytokine secretion assay

Cytokine secretion log2FC secretion data from THP-1 macrophages exposed to PFAS was analyzed using the BMDx software [35]. Cytokine secretion profiles were first filtered using an ANOVA threshold of $p < 0.05$ to ensure differential secretion. To assess dose-dependent cytokine expression, multiple statistical models were fitted to the cytokine secretion data, including two-parameter exponential (exp2), Hill, linear, two-parameter log-logistic (llog2), two-parameter Michaelis-Menten (mm2), second-degree polynomial (poly2), power, and Weibull (Weibull12) models. Fitted models with an R-squared (R^2) value of at least 0.6 were retained and the best-fitting model for each cytokine was selected based on the lowest Akaike Information Criterion (AIC).

Transcriptomic data processing

Because these technologies differ substantially in measurement principles and data structure, to maximize interoperability of downstream analyses, we adopted a standardized preprocessing framework in which all platforms underwent the same preprocessing steps while implementing platform-specific procedures required for each technology. Each dataset was processed independently, and in cases where an entry contained experiments from multiple organisms, data were further separated by organisms to avoid introducing artificial batch effects across species.

RNA-seq and TempO-seq preprocessing of publicly available and generated data

Raw sequencing reads were downloaded from the European Nucleotide Archive (ENA) for all RNA-seq datasets. Sample quality was first assessed using FastQC (v0.12.1) [93]. When individual samples were sequenced across multiple lanes, the corresponding FASTQ files were merged prior to further processing. Reads were trimmed with Cutadapt (v4.4) to remove adapter sequences and low-quality bases (Phred < 20) [94]. Only reads longer than 60 bp after trimming were retained.
Trimmed reads were aligned to the appropriate reference genome using HISAT2 (v2.2.1)[95]: *Mus musculus*: GRCm39 (Ensembl v110), *Rattus norvegicus*: mRatBN7.2 (Ensembl v112), *Danio rerio*: GRCz11 (Ensembl v110), *Homo sapiens:* GRCh38 (Ensembl v110).

Alignment files were converted from SAM to BAM, filtered for uniquely mapped reads, and coordinate-sorted using SAMtools (v1.18) [96]. Raw gene-level counts were then extracted using Rsubread (v2.16.1) with the Ensembl GTF annotation files matching each reference genome [97]. Count matrices were processed using a standardized workflow in RStudio with DESeq2 (v1.36.0) [98]. Lowly expressed genes were removed using a wrapper of the filtered.data function from NOISeq (v2.31.0) [99], employing the proportion test to evaluate whether a gene's relative expression significantly exceeded a CPM/10^6 threshold. Genes with adjusted *p*-values > 0.05 across all conditions were excluded. This filtering step improves both statistical power and biological interpretability by removing genes with insufficient signal. Filtered datasets underwent quality assessment via PCA of variance-stabilizing transformed (VST) counts to confirm sample consistency and detect potential outliers. Normalization was performed using the median-of-ratios method implemented in DESeq2 [98], which accounts for differences in library size. Differential expression analysis was conducted using the DESeq2 Wald test, with independent filtering enabled to optimize detection power [98] [REF]. For all datasets, treated samples (stratified by chemical, dose, and timepoint) were compared to their matched controls. *P*-values were adjusted using the Benjamini-Hochberg false discovery rate (FDR) procedure. The full list of differentially expressed genes was also filtered to only contain significantly differentially expressed genes with the threshold of |logFC| > 0.58 and Benjamini & Hochberg adjusted p-value < 0.05.

TempO-Seq datasets were processed using a workflow aligned with the RNA-seq pipeline, with adaptations specific to the characteristics of the TempO-Seq platform. Raw count matrices were retrieved directly from GEO. In contrast to RNA-seq data, TempO-Seq counts represent probe-level measurements, with multiple probes potentially mapping to the same gene. To obtain gene-level expression values, probes annotated to the same gene were aggregated by computing their median count. Following probe aggregation, lowly expressed features were filtered using filtered.data function from the NOISeq package [99]. Quality assessment was performed through PCA of variance-stabilized (VST) transformed data,

using DESeq2 package and samples flagged as outliers were excluded. Additionally, samples with fewer than 300,000 total counts, a threshold indicative of insufficient sequencing depth, were removed. Normalization and differential expression analysis were then conducted using the DESeq2 package, following the workflow applied to RNA-seq data described above. Where available, batch covariates were incorporated into the DESeq2 design formula to account for potential confounding structure. For dataset GSE248251, normalization and downstream analysis were performed independently for each species and mouse strain. Raw count matrices, unfiltered and filtered differentially expressed gene files for each entry are provided and are accessible from here 10.5281/zenodo.17985509.

Microarray preprocessing

Microarray datasets generated on Agilent and Affymetrix platforms were preprocessed in R using standardized workflows consistent with those implemented in EUTOPIA software that use state-of-the-art preprocessing pipelines suitable for both platforms variation [46]. Quality control (QC) was first performed using platform-specific steps: *arrayQualityMetrics* for Agilent arrays and *affyQCreport (v1.66.0)* together with *yaqcaffy* (v1.48.00) for Affymetrix arrays [100]. QC outputs, complemented by visual inspection of density distributions, bar plots, dendrograms, and multidimensional scaling plots, were used to identify low-quality or outlier samples, which were removed prior to downstream processing. Following QC, probe filtering was applied to eliminate low-intensity probes: for Agilent arrays, filtering was based on assessing probe robustness relative to negative controls and applying quantile-derived thresholds tailored to the structure of each dataset and replicate number. After filtering, signal intensities were $\log_2$-transformed and normalized. Quantile normalization implemented in the *limma* package (v3.54.2) was used for Agilent arrays, while the *justRMA* function from the *affy* package (v1.76) was applied to Affymetrix arrays [101,102]. The resulting normalized expression matrices are included in the final data collection. Batch effects were evaluated through principal component analysis and hierarchical clustering, to detect potential technical variation [46]. Unknown sources of variation were further assessed using surrogate variable analysis via the *sva* package (v3.46) [103]. If variation associated to known technical variables or any of the surrogate variables was observed, its correlation with biological variables of interest was assessed via a confounding plot. Probe annotation was performed using the most updated resources available for each platform. For Agilent arrays, annotation files corresponding to the custom array design used were obtained from the Geo accession page and probes were mapped to Ensembl gene identifiers. For Affymetrix arrays, annotations were derived from the latest custom CDF files from BrainArray (http://brainarray.mbni.med.umich.edu/Brainarray/Database/CustomCDF/CDF_download.

asp, 2024)[104], providing gene-level mappings to Ensembl IDs. Differential expression analysis was carried out using the *limma* package. Comparisons were made between each specific experimental group consisting of a single chemical exposure, dose, time point, biological system and organism and its corresponding control samples. As a result of the differential expression analysis, we provide within the collection for each entry full lists of genes with their specific fold changes and statistics as well as the results filtered to only contain significantly differentially expressed genes with the threshold of $|logFC| > 0.58$ and Benjamini & Hochberg adjusted p-value < 0.05.

Molecular Docking

Molecular docking analyses were performed using Maestro (Schrödinger Suite 2025) to estimate the binding affinities of PFAS to selected nuclear receptors (NR). Ligand structures were preprocessed with LigPrep (Schrödinger), using the OPLS4 force field to generate low-energy conformers, assign proper ionization states at pH 7.4 ± 0.5, and standardize molecular geometries[39,40]. Protein targets included PPAR-α (PDB IDs: 2P54, 2Q5S), PPAR-γ (2A0Z), PPAR-β/δ (3TKM), and PXR (6DUP). Because no experimentally resolved structure is available for SXR, its AlphaFold-predicted model was used. All protein structures were preprocessed with the Protein Preparation Wizard to assign protonation and tautomeric states at pH 7.4, optimize hydrogen-bonding networks, and perform restrained minimization under the OPLS4 force field. Ligand-binding sites were identified using SiteMap, and receptor grids were generated for each target based on the highest-ranked predicted site. Docking was performed in Glide using Standard Precision mode with default scoring functions, including rewards for hydrogen-bond formation[39,40]. Docking scores (GlideScores) were obtained under identical conditions to ensure comparability across targets and were used to approximate binding affinity, with more negative values indicating stronger predicted interactions. Binding affinity results were visualized with the ComplexHeatmap package[41].

Prioritization of relevant PFAS-induced liver steatosis genes

To identify genes specifically associated with PFAS-induced liver steatosis across biological systems, we used the aggregated KE enrichment results that also formed the basis for AOP interpretation and KE–KE network reconstruction. First, the 124 unique KEs were divided into two groups: (i) *steatosis-relevant KEs*, defined as those included in AOPs explicitly linked to the AO "Increased, Liver Steatosis," and (ii) *non-steatosis KEs*, comprising all remaining events. Each group was then further divided by biological system. For each of the 124 KEs, we collected all genes contributing to its enrichment. A gene relevance score was calculated

as the proportion of PFAS exposure studies in which the gene contributed to significant enrichment of that KE, normalized to the total number of studies available for that system. This scoring approach provided a measure of the consistency with which each gene was associated with a given biological response. Within each biological system, we then statistically compared the distribution of relevance scores for each gene between steatosis-relevant and non-steatosis KEs using a two-sided *t*-test. P-values were adjusted for multiple testing using the Benjamini–Hochberg False Discovery Rate (FDR) procedure. For each biological system, genes were considered significantly associated with PFAS-induced liver steatosis when their adjusted FDR was below or equal to 0.05 and their mean relevance score in steatosis-relevant KEs exceeded 0.8 (i.e., present in ≥80% of contributing studies).

Supplementary results and discussion

Different PFAS congeners reduce cell viability at varying concentrations

To gain molecular mechanistic insights into how newer PFAS compounds, which toxicological characterization is still fragmented, influence immune responses, particularly in comparison to their legacy counterparts, we exposed macrophages like THP-1 to seven PFAS congeners selected among legacies and newer alternatives that are gaining attention for their effect of immune systems. We first exposed THP-1 to each PFAS in a wide range of concentrations to draw cytotoxicity curves over 72h. This step guided the selection of appropriate subtoxic equipotent exposure concentrations (IC5, IC10 and IC20, Table S6) that could be used to compare functional and transcriptional responses across the compounds. Beyond the selection of subtoxic concentrations this step provided information on the comparative cytotoxicity of the compounds on macrophages. Each of the seven PFAS reduced cell viability in a concentration-dependent manner within the tested range (Figure S11). Based on the 72h dose-response curves and the corresponding IC10 values, the cytotoxicity rank was: PFESA BP2>PFESA BP1>8:2 Cl-PFESA>PFOA>PFOS>DOPFLCA>PFHpS.

Previous studies have shown that PFAS toxicity is often influenced by chain length, with longer perfluorinated backbones generally associated with greater toxicity [105]. In our study, all compounds possessed 7-8 carbon atoms, placing them in comparable range in terms of chain length indicating that the observed differences in potency likely stem from other structural features. Notably, the three most cytotoxic congeners, PFESA-BP1, PFESA-BP2, and 8:2 Cl-PFESA, are all ether-containing sulfonic acids, suggesting that ether linkages may enhance macrophage toxicity within this compound class. While sulfonic acids are typically

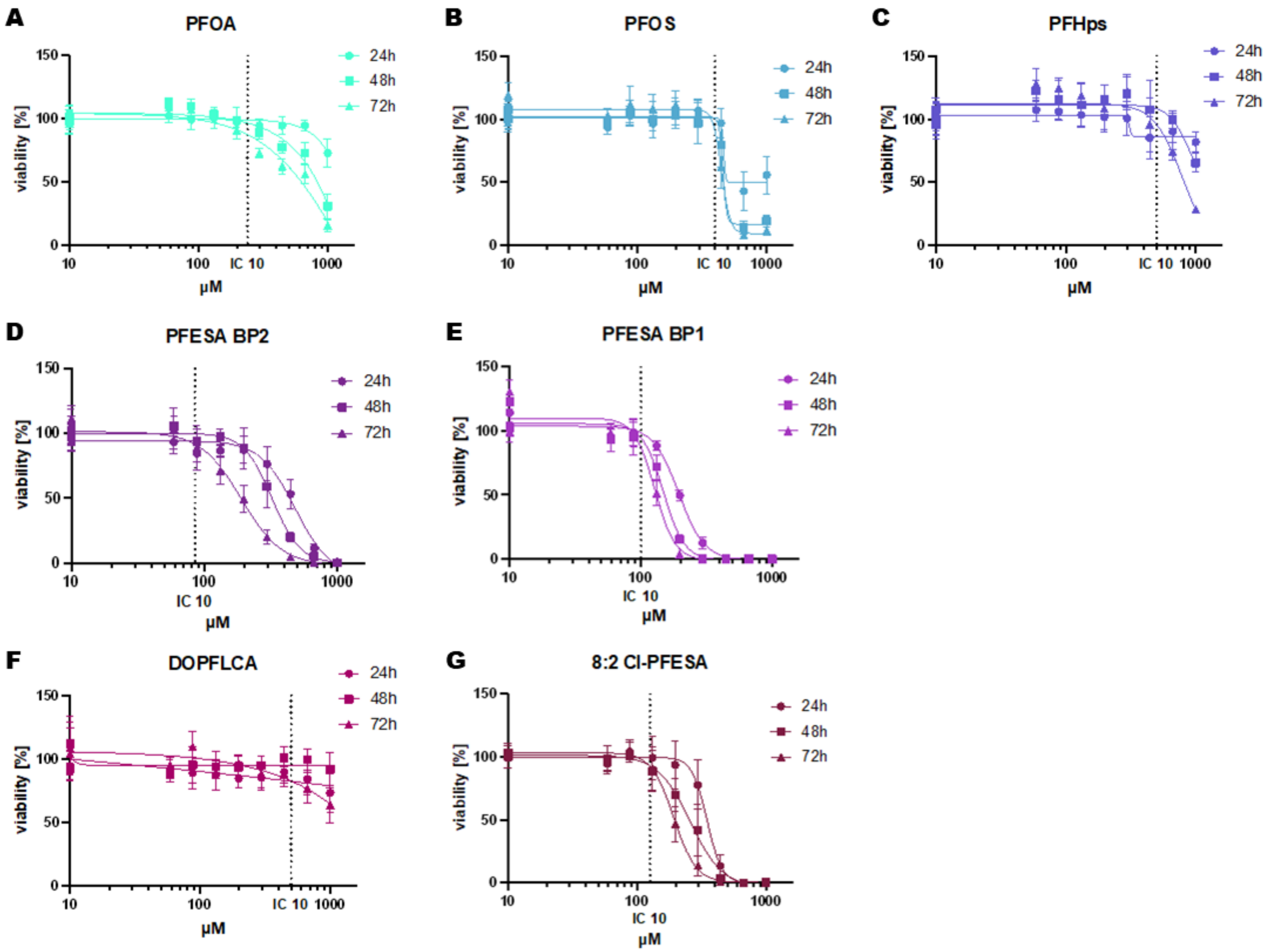

Figure S11. Cell viability curves following the exposure of THP-1 macrophages to the PFAS congeners for 24h, 48h and 72h. For each chemical, 8-point dose-response curve was determined using 4PL curve fitting. In the y-axis, percentage change from 0 dose is represented while the x-axis denotes the dose from 0 to 1000 µM. Each datapoint is represented as mean (+- standard deviation) using 7 parallel datapoints. A) PFOA, B) PFOS, C) PFHpS, D) PFESA BP2, E) PFESA BP1, F) DOPFLCA G) 8:2 Cl-PFESA.

considered more toxic than carboxylic acids (ref), our results indicate that ether-containing sulfonic acids exert stronger effects than both non-ether sulfonic acids (e.g., PFOS, PFHpS) and ether and non-ether carboxylic acids (e.g., PFOA, ADONA). This deviation from classical BEHAVIOR suggests a cell type–specific response, consistent with prior reports demonstrating that PFAS-induced cytotoxicity varies substantially across cell types

[106,106]. Importantly, these findings reveal that emerging PFAS alternatives, particularly ether sulfonates, can elicit stronger cytotoxic effects on macrophages than their legacy counterparts. This highlights that current PFAS replacement strategies do not necessarily reduce biological activity.

PFAS exposures modulate cytokine secretion in a dose-dependent manner

Previous studies have shown that PFAS are capable of eliciting an immunogenic response through the secretion of pro-inflammatory cytokines. PFOS and PFOA induce IL-1β, TNF-α, and IL-6 in macrophages with the AIM2 inflammasome sensor implicated in signal transduction [107]. PFAS also have immunomodulatory effects, altering the circulating immune cell subpopulations in peripheral blood mononuclear cells (PBMCs) and muting vaccination specific antibody responses [108,109].

However, limited studies have comprehensively assessed immune cell responses when directly comparing legacy and next generation PFAS exposures, leaving significant gaps in our mechanistic understanding of their immunomodulatory potential. Recent work in zebrafish larvae by Yao et al. (2024) screened four PFAS, including legacy compounds and newer-generation alternatives, reporting comparable immunotoxic effects across both classes [110]. Epidemiological evidence also supports the role of both legacy and emerging PFAS in disrupting cytokine homeostasis in human populations [111]. As real-life exposures typically occur as complex mixtures, dissecting the immunomodulatory effects of individual PFAS types remains critical. Such mechanistic understanding can enable the identification of particularly hazardous PFAS and supports efforts to compare and regulate distinct chemical exposures more effectively.

To further investigate and compare the immunomodulatory effects of legacy and next-generation PFAS, we measured cytokine secretion in the culture medium of THP-1 macrophages exposed to equipotent, increasing concentrations of seven PFAS compounds (Table S6). Cytokine levels were quantified after 24, 48, and 72 hours of exposure.

Whenever a dose-dependent cytokine change was observed, it occurred in an increasing direction, with only a single instance of dose-dependent downregulation (blue triangle), indicating a pro-inflammatory cytokine response to PFAS exposure irregardless of the PFAS type (Figure S12). Among the tested PFAS, the legacy compounds PFOA, PFOS, and PFHpS elicited the most extensive dose-dependent increases in cytokine secretion, suggesting stronger immunomodulatory effects compared to the next-generation (poly-)ether PFAS. PFOA consistently upregulated all tested cytokines at least at one measured timepoint. PFOS and PFHpS similarly exhibited dose-dependent increases in 7 out of 10 and 9 out of 10 cytokines, respectively (Figure S12). PFOS and PFHpS induced the strongest increases in IL-

10, IL-1β, and IL-8 levels. These cytokines are key mediators of inflammation and immune regulation, once again showing that these PFAS compounds are capable of significantly impacting inflammatory pathways [106, 109].

In contrast, the next-generation (poly-)ether PFAS, including PFESA BP2, PFESA BP1, DOPFLCA, and 8:2 Cl-PFESA, exhibited fewer dose-dependent cytokine changes, indicating more limited cell activation (Figure S12). Among these compounds, DOPFLCA produced the most notable cytokine activation, though still less extensive than that induced by the legacy PFAS. PFESA BP2 demonstrated a particularly limited inflammatory response, with dose-dependent induction observed only for TNF-α and IL-8 at a single early timepoint. This minimal proinflammatory cytokine activation suggests decreased immunomodulatory effect of new-generation PFAS.

Concluding from the cytokine secretion profiles in our results, all PFAS tested were capable of activating macrophages by inducing the secretion of pro-inflammatory cytokines. Legacy PFAS induced a stronger proinflammatory response in macrophages compared to next-generation PFAS, confirming their established immunotoxic effects [107]. The next-generation PFESA-BP2 was the only PFAS which didn't cause an extensive pro-inflammatory response.

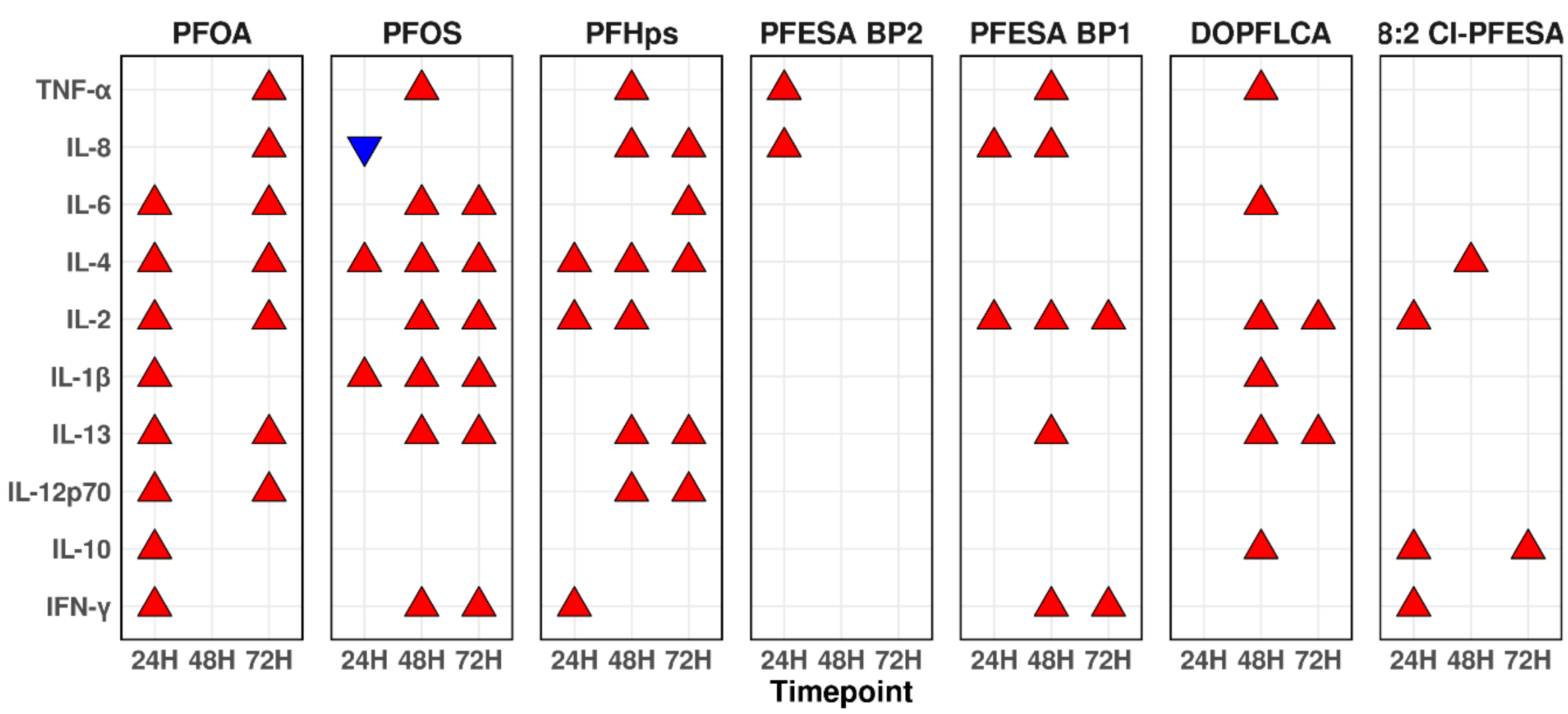

Figure S12. Dose-dependent secretion of proinflammatory cytokines in THP-1 cells exposed to multiple doses of PFAS at 24, 48, and 72 hours. Each panel represents a different PFAS compound. The y-axis lists cytokines measured, while the x-axis represents the timepoints. Red triangles indicate cytokines with a dose-dependent increase in secretion, while blue triangles indicate cytokines with a dose-dependent decrease in secretion.

AOP annotation and omics data highlight a panel of genes representing the core mechanism of PFAS-induced hepatotoxicity

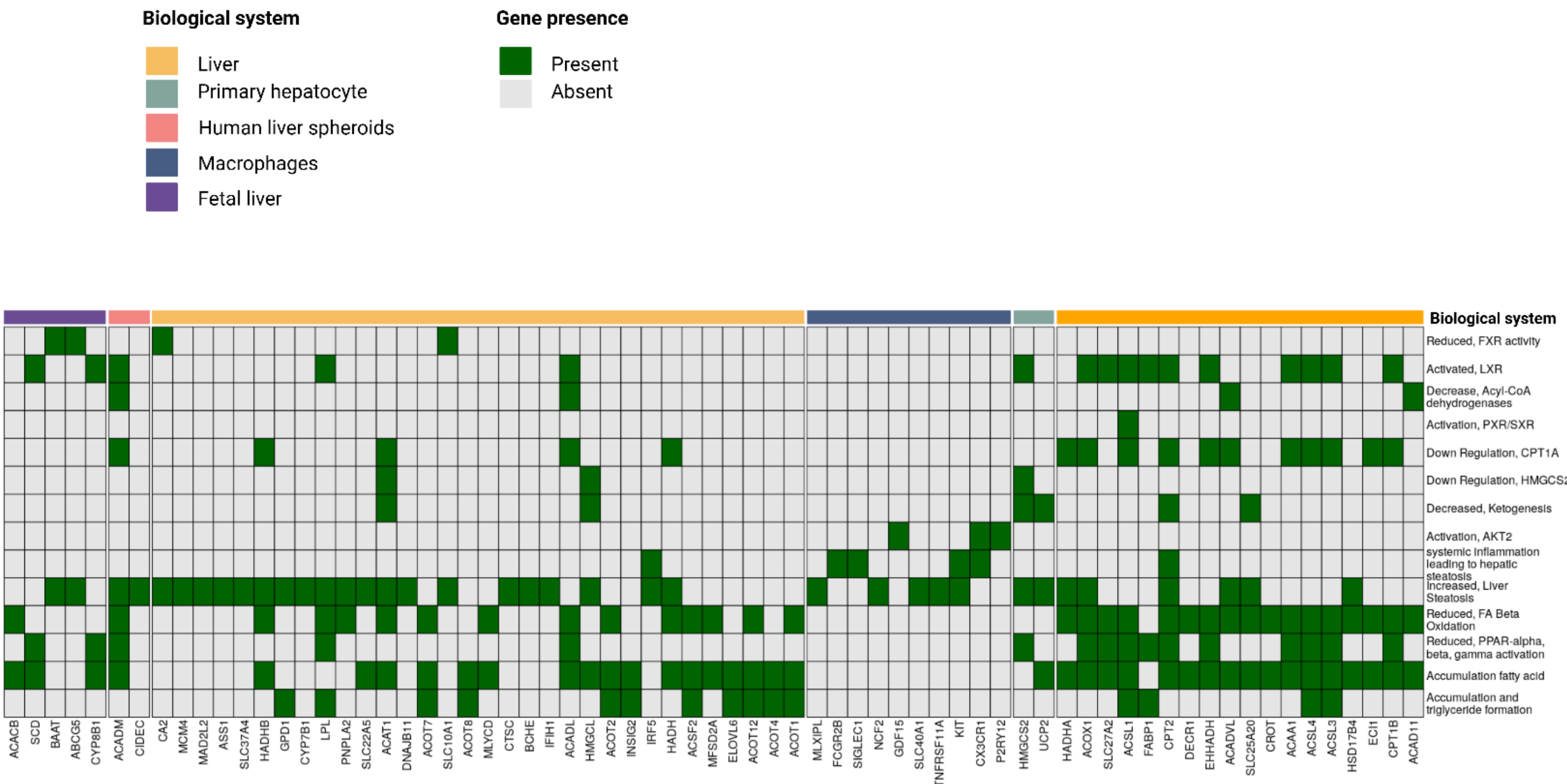

Figure S13. Gene-key event mapping for PFAS-induced liver steatosis. The heatmap displays the presence-absence matrix of genes selected as relevant for the PFAS-induced liver steatosis mechanism. Columns represent individual genes, and rows correspond to KEs implicated in the steatosis AOP network. A green cell indicates that a gene is associated with (i.e., mapped to) a given KE, whereas grey indicates absence of association. The annotation bar above denotes the biological system in which each gene was identified, highlighting context-specific gene-KE association across liver models.

We observed that the mechanism underlying PFAS-induced liver steatosis is characterized by context-dependent chains of key events that converge into a conserved mechanistic core across different biological systems. This indicates that, despite system-specific molecular perturbations, the apical events driving liver steatosis are preserved and could serve as predictive markers of PFAS-induced hepatotoxicity. Given the urgent need to characterize PFAS as a chemical class, amid a risk assessment process that remains slow, resource-intensive, and ethically challenging, we leveraged our AOP-based framework to derive a

panel of relevant genes for the PFAS-induced steatosis mechanism. For each biological system, we prioritized genes that were both frequently altered across PFAS exposures and specifically associated with steatosis-related events. To this end, we compared the distribution of gene relevance scores between steatosis-related events and not steatosis-related events. Genes showing statistically higher relevance within the steatosis group (*t*-test, relevance score >80%) were retained, resulting in a panel of 69 genes representing the most consistent molecular markers of the PFAS-induced steatosis mechanism. This panel includes a set of system specific genes and a set of genes shared across multiple biological systems, with the exception of macrophages, whose relevant genes were system specific. This set of genes covers 14 out of 16 events included in the PFAS-induced liver steatosis mechanism and mostly represents the conserved mechanistic core leading to the adverse outcome (Figure S13). Indeed, the panel includes genes involved in lipid accumulation and biosynthesis (e.g., *SLC27A2*, *CIDEC*, *CYP7B1*, *CROT*, *ACAT1*, *ACSL3*, *ACSF2*, *ELOVL6*, *ACACB*, *CYP8B1*), triglyceride formation (*LPL*, *PNPLA2*, *BAAT*), and mitochondrial fatty acid β-oxidation (e.g., *ACOX1*, *HADHA*, *ACADM*, *CPT2*, *DECR1*, *ACADVL*, *HADHB*, *SLC25A20*, *ACOT8*, *MLYCD*, *ECI1*), all central process to the progression of liver steatosis. Even though macrophages activated a specific chain of events sharing with the core mechanism only the adverse outcome, the half of macrophage-specific genes are associated with the adverse effect, among which *TNFRSF11A*, encoding part of the TNF-α receptor implicated in NAFLD progression, and *MLXIPL*, encoding the ChREBP/Mlx transcriptional complex that regulates lipogenesis, are particularly relevant [112,113]. This pattern of genes relevance confirms the context-dependent activation of the liver steatosis mechanistic branches and further reinforces the suggestion of considering the test system in PFAS risk assessment. Additionally, a large fraction of these genes was specific to liver tissue; however, many of them also contributed to the enrichment of steatosis-related events in primary hepatocyte exposure studies. This overlap suggests that simpler *in-vitro* systems can recapitulate alterations observed in complex in vivo models, effectively capturing the conserved portion of the mechanism. Together these findings indicate that the identified set of PFAS liver steatosis-relevant genes could serve as a biologically grounded, rapid and cost-effective tool for screening PFAS toxicity, providing early insights into their potential to induce steatosis and indicating when further targeted testing is warranted. The same strategy can also be applied during the design of PFAS alternatives, allowing preliminary toxicity evaluation already in early development phases.
